# USP12 translocation maintains interferon antiviral efficacy by inhibiting CBP acetyltransferase activity

**DOI:** 10.1371/journal.ppat.1008215

**Published:** 2020-01-03

**Authors:** Jin Liu, Lincong Jin, Xiangjie Chen, Yukang Yuan, Yibo Zuo, Ying Miao, Qian Feng, Hongguang Zhang, Fan Huang, Tingting Guo, Liting Zhang, Li Zhu, Feng Qian, Chuanwu Zhu, Hui Zheng

**Affiliations:** 1 International Institute of Infection and Immunity, Institutes of Biology and Medical Sciences, Soochow University, Suzhou, China; 2 Jiangsu Key Laboratory of Infection and Immunity, Soochow University, Suzhou, China; 3 The Affiliated Infectious Diseases Hospital of Soochow University, Suzhou, China; The University of Chicago, UNITED STATES

## Abstract

CREB-binding protein (CBP) participates in numerous transcription events. However, cell-intrinsic inhibitors of CBP are poorly defined. Here, we found that cellular USP12 interacts with the HAT domain of CBP and inhibits CBP’s acetyltransferase activity. Interestingly, USP12 positively regulates interferon (IFN) antiviral signaling independently of its deubiquitinase activity. Furthermore, we found that in IFN signaling USP12 translocates from the cytoplasm to the nucleus. The decrease in cytoplasmic USP12 facilitates CBP-induced acetylation and activation of IFN signaling proteins in the cytoplasm. Moreover, USP12 accumulation in the nucleus blocks CBP-induced acetylation of phosphorylated STAT1 (p-STAT1) and therefore inhibits the dephosphorylation effects of TCPTP on p-STAT1, which finally maintains nuclear p-STAT1 levels and IFN antiviral efficacy. USP12 nuclear translocation extends our understanding of the regulation of the strength of IFN antiviral signaling. Our study uncovers a cell-intrinsic regulation of CBP acetyltransferase activity and may provide potential strategies for IFN-based antiviral therapy.

## Author summary

Activated p-STAT1 is a determinant for the strength of IFN antiviral signaling. We and other groups have demonstrated that activated p-STAT1 is regulated by multiple protein post-translational modifications, including phosphorylation, acetylation and ubiquitination. In this study, we revealed that CBP-mediated acetylation regulation of p-STAT1 is modulated by the deubiquitinase USP12 in a deubiquitinase activity-independent manner. USP12 translocates into the nucleus in IFN signaling, which critically regulates nuclear p-STAT1 levels and IFN antiviral activity by inhibiting CBP’s acetyltransferase activity. Importantly, we demonstrated that USP12 is a cell-intrinsic inhibitor of the acetyltransferase CBP. These findings promote the understanding of delicate regulation of both CBP-mediated acetylation and IFN antiviral signaling.

## Introduction

Protein acetylation has recently attracted much attention due to its broad-spectrum and essential roles in regulating cellular functions. As an evolutionarily conserved post-translational modification (PTM) of proteins, protein acetylation is catalyzed by at least five distinct families of acetyltransferases [1–7]. Among these acetyltransferases, the CREB-binding protein (CBP) and its homologue P300 are ubiquitously expressed in most types of cells in not only all mammals but also flies, worms and plants [8, 9]. Recent time-resolved acetylome analyses have revealed the rapid dynamics and broad scope of CBP/P300 acetylation reactions at thousands of sites [10]. CBP and P300 can regulate over 400 transcription factors and numerous regulatory proteins [11], which makes CBP/P300 a major regulation of essential cellular activities, such as proliferation, cell cycle, differentiation, apoptosis and DNA damage responses [12].

Despite closely related features in structure and function, CBP and P300 can play distinct roles in cellular activities [13]. CBP contains several major domains that control either the interaction with different proteins or the acetyltransferase activity of CBP. CBP localizes mostly in the nucleus to regulate transcription events by acetylating a large number of transcription-related factors [11, 14, 15]. For example, in interferon (IFN) signaling pathway, nuclear CBP can acetylate tyrosine 701-phosphorylated STAT1 (pY701-STAT1). Acetylation of pY701-STAT1 results in the recruitment of the phosphatase TCPTP, which in turn downregulates the levels of nuclear pY701-STAT1 and inhibits transcriptional expression of Interferon-stimulated genes (ISGs) [16]. During DNA damage response, CBP can acetylate p53 and other proteins for transcription regulation [17, 18]. The central roles of CBP in regulating cellular functions have spurred efforts to develop specific inhibitors of the acetyltransferase CBP in preclinical studies [19]. In particular, a small molecule inhibitor (ICG-001) blocks the binding of CBP to β-catenin, decreases tumorigenic phenotypes and promotes drug sensitivity in tumors [20, 21]. However, to our knowledge, cell-intrinsic inhibitors of the acetyltransferase activity of CBP have not been identified thus far.

The regulation of the acetyltransferase activity of CBP remains largely unexplored. Several reports have demonstrated the influence of CBP PTMs on its acetyltransferase activity. Phosphorylation of CBP has been shown to affect CBP acetyltransferase activity in cell-cycle progression [22, 23]. CBP methylation could enhance its acetyltransferase activity in estrogen receptor signaling [24]. Apparently, these PTMs produce a relatively stable activity status of CBP in cells. Given that CBP mediates the acetylation of a multitude of cellular proteins, the stable CBP activity status could be disadvantageous to the differential regulation of various intracellular signaling pathways. Thus, one could ask whether CBP activity undergoes more flexible regulations to adapt to the rapid switch between inhibition and activation of signaling pathways.

In our mass spectroscopy analysis, we noticed an interaction between USP12 and CBP. USP12 belongs to the ubiquitin-specific protease family of deubiquitinases (DUBs). It has been reported that USP12 can upregulate levels of several proteins by its deubiquitinase activity [25–27]. Interestingly, we found that USP12 did not affect CBP protein levels, suggesting a catalytic activity-independent regulation by USP12. Intriguingly, we found that USP12 significantly inhibited CBP-mediated pan-acetylation effects in cells. Furthermore, our results demonstrated that USP12 could be a cellular inhibitor of the acetyltransferase CBP.

Based on these findings, we sought to determine the significance and mechanism of the USP12/CBP interaction. Given that CBP regulates both the cytoplasmic signaling and the nuclear transcription factor STAT1 in IFN signaling, we further focused on studying the roles of the USP12/CBP interaction in IFN signaling. We found that IFN signaling stimulated translocation of USP12 from the cytoplasm to the nucleus. The decrease in cytoplasmic USP12 facilitates CBP-mediated acetylation of IFN signaling proteins. The accumulation of USP12 in the nucleus blocks the acetylation effect of CBP on pY701-STAT1, thus inhibiting the binding and dephosphorylation effects of TCPTP on nuclear pY701-STAT1. As a consequence, USP12 translocation from cytoplasm to nucleus is critical for both activation and sustainment of IFN signaling. Our findings could provide new strategies for inhibiting CBP acetyltransferase activity in cells and for improving IFN-based antiviral therapy.

## Results

### USP12 interacts with CBP and blocks the acetyltransferase activity of CBP

**Fig 1:**
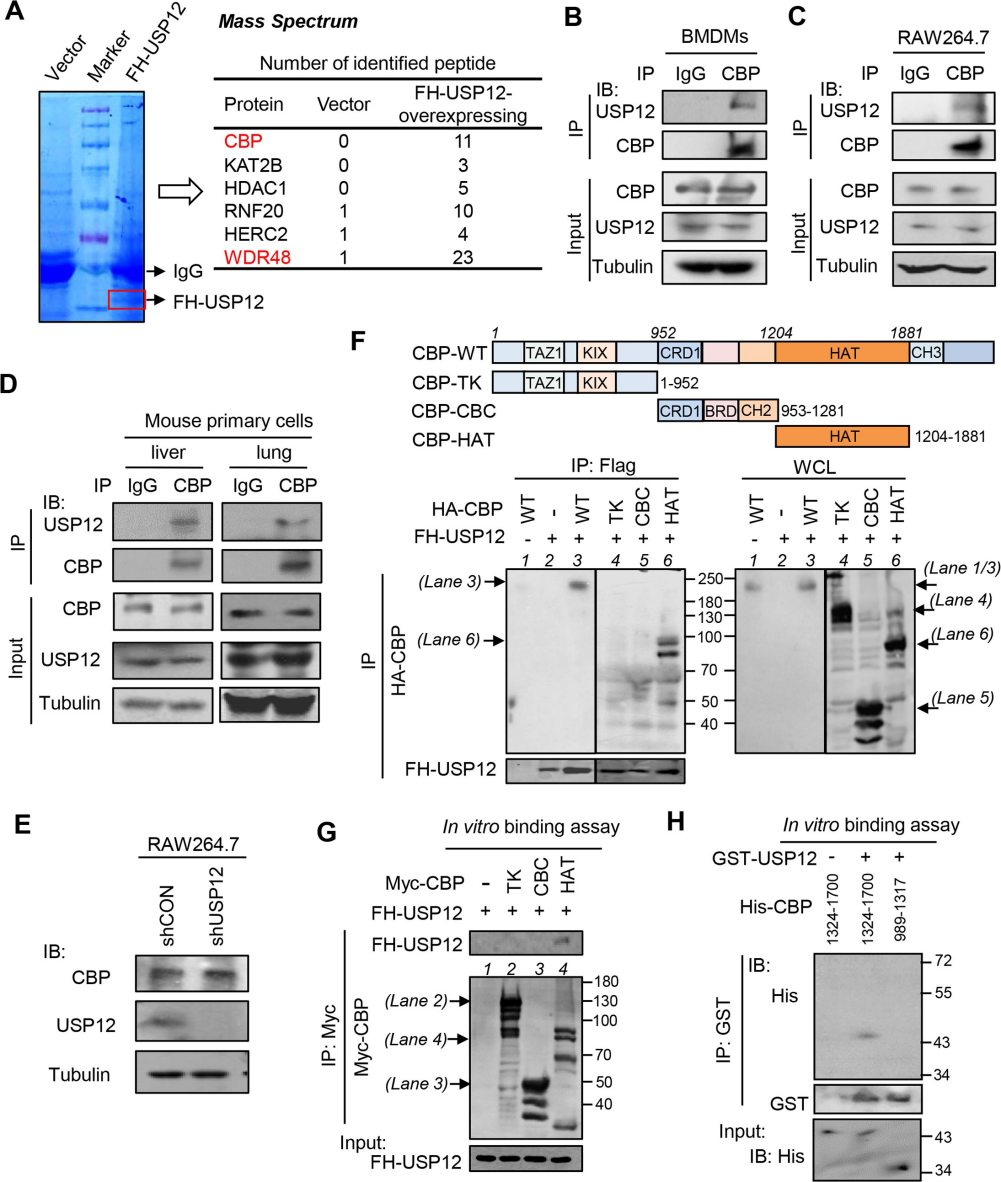
USP12 interacts with the HAT domain of CBP. (A) HEK293T cells were transfected with empty vectors or Flag-HA-USP12 (FH-USP12). The whole cell lysates (WCL) were subjected to immunoprecipitation using Flag (M2) beads. The interacting proteins were analyzed by mass spectrometry (N = 2). (B, C) Immunoprecipitation analysis of the interaction between endogenous USP12 and CBP in BMDMs (B) and RAW264.7 cells (C). (D) Immunoprecipitation analysis of the interaction between endogenous USP12 and CBP in mouse primary lung and liver cells. (E) Western blot analysis of endogenous CBP levels in RAW264.7 cells transfected with either control shRNAs (shCON) or shRNAs against USP12 (shUSP12). (F) The interaction analysis in HEK293T cells cotransfected with FH-USP12 and HA-CBP wild-type (WT) or Myc-CBP mutants including CBP-TK (1–952), CBP-CBC (953–1281), CBP-HAT (1204–1881) as indicated. (G) *In vitro* binding assay for analysis of the interaction between FH-USP12 and Myc-CBP mutants, including CBP-TK (1–952), CBP-CBC (953–1281), CBP-HAT (1204–1881), which were immunoprecipitated from HEK293T cells transfected with the corresponding plasmids. (H) *In vitro* binding assay for analysis of the interaction between bacterially expressed GST-USP12 proteins and His-CBP mutant proteins, including CBP-HAT (1324–1700) and CBP-CBC (989–1317), which were purified from E. coli.

To explore the role of USP12 in innate antiviral immunity, we first performed mass spectrometry analysis to identify the potential binding proteins of USP12. We noticed some PTM-related proteins in the mass spectrometry data (Fig 1A). Among them, the acetyltransferase CBP attracted our attention. Thus, we further confirmed the interaction between USP12 and CBP. Flag-HA tagged USP12 (FH-USP12) can bind with HA-CBP (S1A Fig) or endogenous CBP (S1B Fig). In bone marrow-derived macrophages (BMDMs) isolated from mice and RAW264.7 cells, endogenous USP12 also interacted with endogenous CBP (Fig 1B and 1C). Importantly, USP12 constitutively binds with CBP in mouse primary lung and liver cells (Fig 1D). Given that USP12 is a member of the deubiquitinases, we speculated that USP12 could upregulate CBP protein levels. However, we found that either knockdown (Fig 1E) or overexpression (S1C Fig) of USP12 did not noticeably affect CBP protein levels. To further determine the USP12-mediated regulation of CBP, we analyzed the key interacting domain of CBP with USP12. Interestingly, USP12 can interact with the HAT domain of CBP. Deletion of CBP’s HAT domain abolished the binding of USP12 (Fig 1F). *In vitro* binding assay further demonstrated that USP12 binds with the HAT domain of CBP (Fig 1G). Using bacterially expressed USP12 and CBP proteins, we also confirmed the interaction between USP12 and the HAT domain of CBP (Fig 1H). Together, these findings demonstrated that USP12 constitutively binds with CBP in cells.

**Fig 2:**
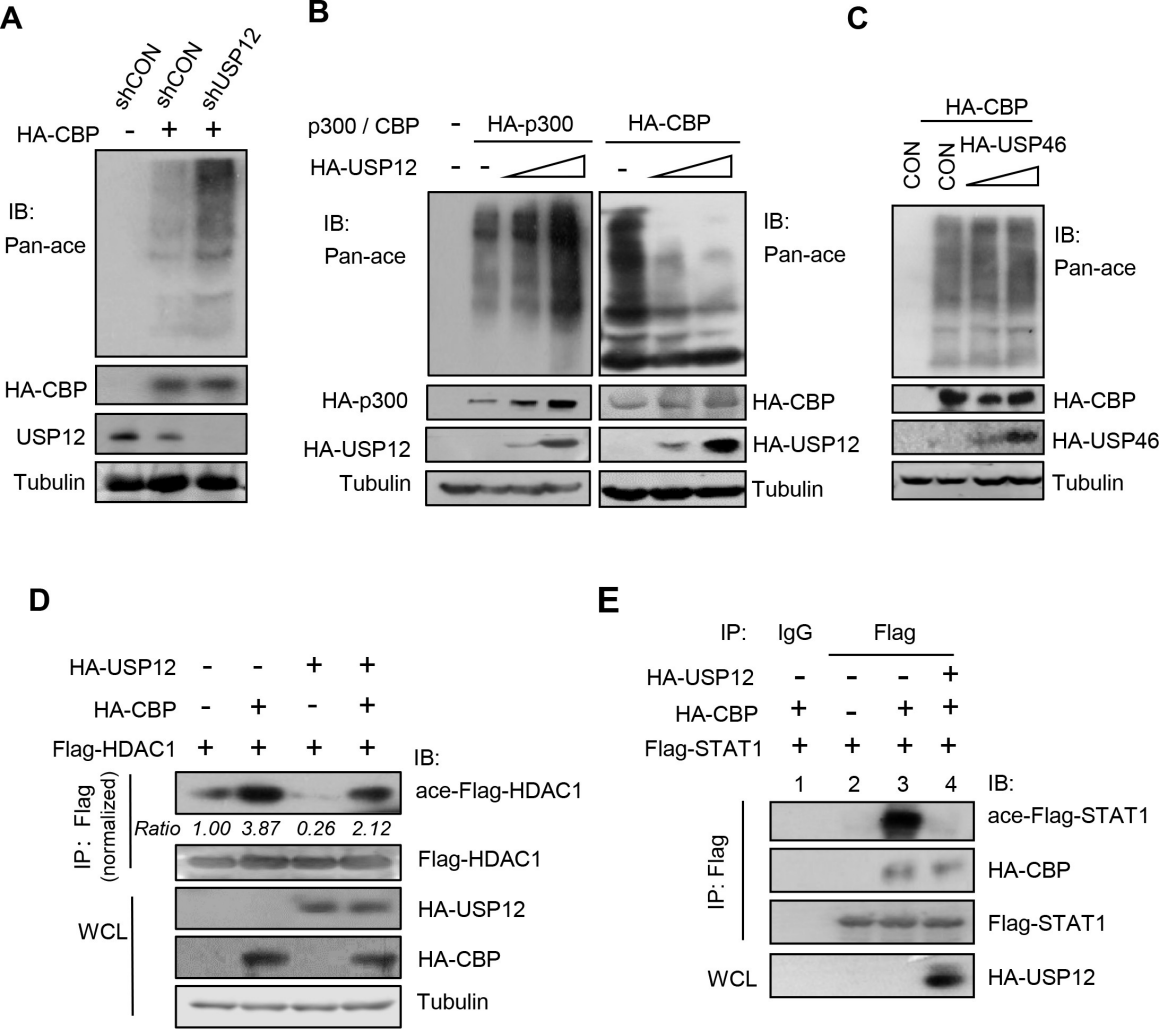
USP12 blocks the acetyltransferase activity of CBP. (A) Western blot analysis of pan-acetylation (Pan-ace) levels in HEK293T cells cotransfected with HA-CBP, together with either control shRNAs (shCON) or shRNAs against USP12 (shUSP12). (B) Western blot analysis of pan-acetylation levels in HEK293T cells cotransfected with either HA-P300 (left) or HA-CBP (right), together with empty vectors (CON) or increasing amounts of HA-USP12. (C) Western blot analysis of pan-acetylation levels in HEK293T cells cotransfected with HA-CBP and (or) HA-USP46. (D) Immunoprecipitation analysis of Flag-HDAC1 acetylation in HEK293T cells cotransfected with Flag-HDAC1, HA-CBP, and (or) HA-USP12. (E) Immunoprecipitation analysis of Flag-STAT1 acetylation and the interaction between Flag-STAT1 and HA-CBP in HEK293T cells cotransfected with Flag-STAT1, HA-CBP, and (or) HA-USP12. ace-, acetylated.

Given that the HAT domain harbors the acetyltransferase activity of CBP, we speculated that USP12 could affect CBP’s acetyltransferase activity by binding with its HAT domain. To address this possibility, HA-CBP was overexpressed in cells. We found that HA-CBP promoted cellular pan-acetylation levels, which were further enhanced by USP12 knockdown (Fig 2A), suggesting that cellular USP12 could inhibit CBP-mediated acetylation. In line with this observation, overexpression of USP12 restricted CBP-mediated pan-acetylation in a dose-dependent manner (Fig 2B, right). It is well known that p300 exerts the similar action with CBP in acetylation regulations. However, USP12 overexpression did not reduce pan-acetylation of cellular proteins induced by p300 (Fig 2B, left). Interestingly, we noticed that overexpression of USP12 upregulated p300 protein levels (Fig 2B, left), while knockdown of USP12 inhibited the expression of endogenous p300 proteins (S2A Fig). We further found that FH-USP12 can interact with HA-p300 in cells (S2B Fig). Importantly, USP12 removed ubiquitination of endogenous p300 proteins (S2C Fig). These findings suggest that differently from the role of USP12 in regulating CBP, USP12 could be a deubiquitinase of p300. In addition, overexpression of USP46, which shares more than 90% amino acid sequences identity with USP12, did not inhibit the cellular pan-acetylation induced by CBP (Fig 2C). Furthermore, we found that CBP-mediated acetylation of other proteins, including HDAC1 (Fig 2D) and STAT1 (Fig 2E), was also blocked by USP12 overexpression. Taken together, these findings suggest that USP12 binds to the HAT domain of CBP and blocks CBP’s acetyltransferase activity in cells.

### USP12 inhibits CBP-mediated acetylation and activation of IFN signaling proteins in the cytoplasm

**Fig 3:**
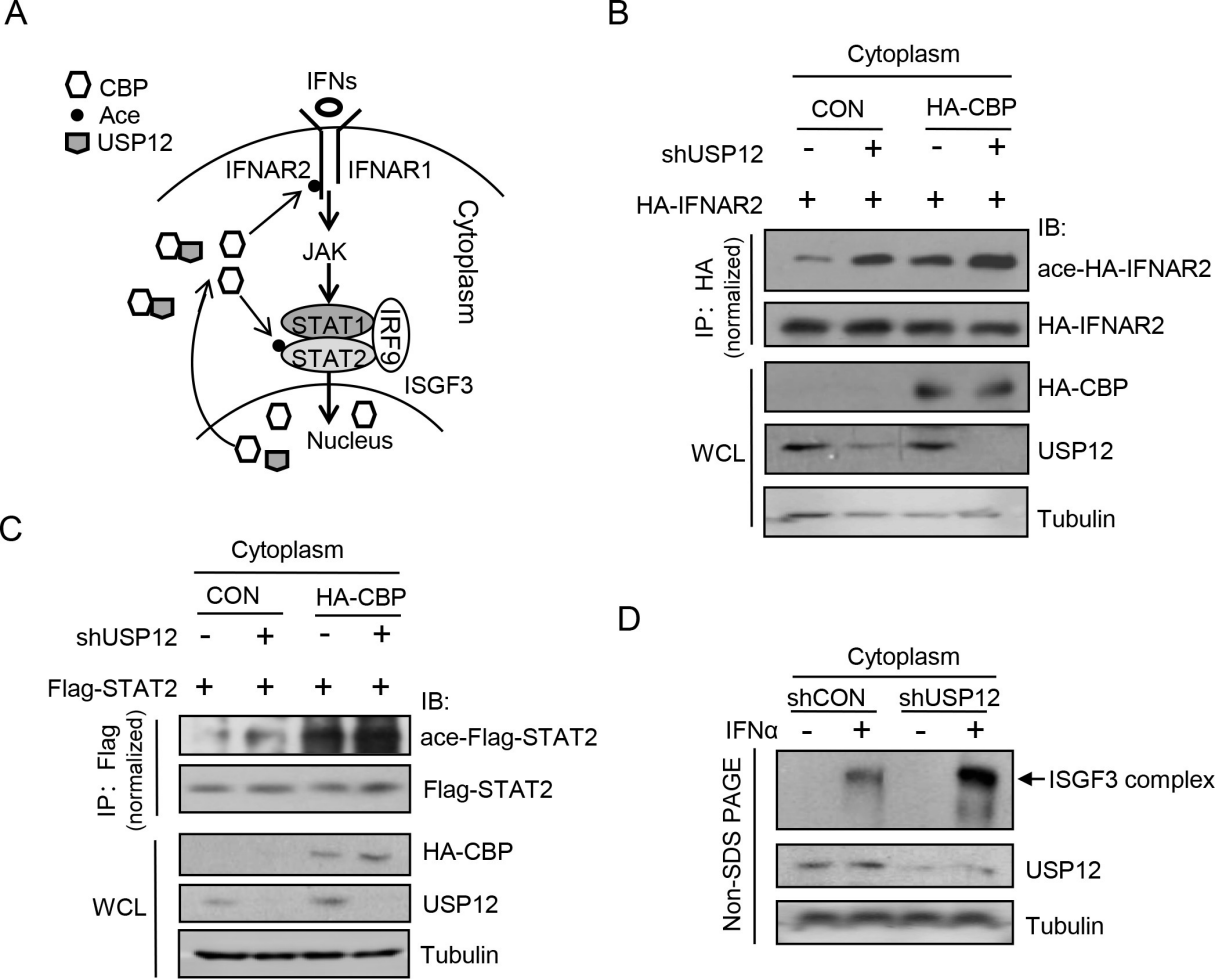
USP12 inhibits CBP-mediated acetylation and activation of IFN signaling proteins in the cytoplasm. (A) The model for CBP cytoplasm translocation and subsequent acetylation of IFN signaling proteins. (B) Immunoprecipitation analysis of IFNAR2 acetylation in the cytoplasm of 2fTGH cells cotransfected with HA-CBP and HA-IFNAR2, together with or without shUSP12 as indicated. (C) Immunoprecipitation analysis of STAT2 acetylation in the cytoplasm of HEK293T cells cotransfected with HA-CBP and Flag-STAT2, together with or without shUSP12. (D) Western blot analysis of ISGF3 complex in the cytoplasm of HEK293T cells transfected with shCON or shUSP12, and then stimulated with IFNα (1,500 IU/ml) for 30 min using native Non-SDS PAGE gels.

To reveal the significance of the USP12/CBP interaction in innate antiviral signaling, we focused on the IFN-induced signaling pathway. CBP has been reported to be located primarily in the nucleus. Upon IFN stimulation, CBP translocates from nucleus to cytoplasm and acetylates some signaling proteins, including IFNAR2 and STAT2, and in turn promotes the formation of the ISGF3 transcriptional complex for IFN signaling activation [28]. Based on this dynamic regulation of CBP, we speculated that USP12 could restrict CBP-mediated acetylation and activation of IFN signaling in the cytoplasm (Fig 3A). Our data showed that knockdown of USP12 promoted acetylation of both HA-IFNAR2 (Fig 3B, left) and Flag-STAT2 (Fig 3C, left). Similarly, when CBP was overexpressed to enhance acetylation modifications of cellular proteins, CBP-mediated acetylation of HA-IFNAR2 (Fig 3B, right) and Flag-STAT2 (Fig 3C, right) in the cytoplasm was upregulated by USP12 knockdown. These results suggest that USP12 could regulate cytoplasmic CBP’s acetylation action and therefore restrict excessive activation of IFN signaling in intact cells. Importantly, in IFN signaling, the formation of the ISGF3 complex in the cytoplasm was significantly enhanced by USP12 knockdown (Fig 3D). Collectively, we think that USP12 is a negative regulator of CBP-mediated activation of IFN signaling in the cytoplasm. Given that the cytoplasm contains both CBP and USP12, one can understand the importance of nuclear CBP translocation to the cytoplasm, which could promote the activation of IFN signaling.

### USP12 translocates from the cytoplasm to the nucleus in IFN signaling

**Fig 4:**
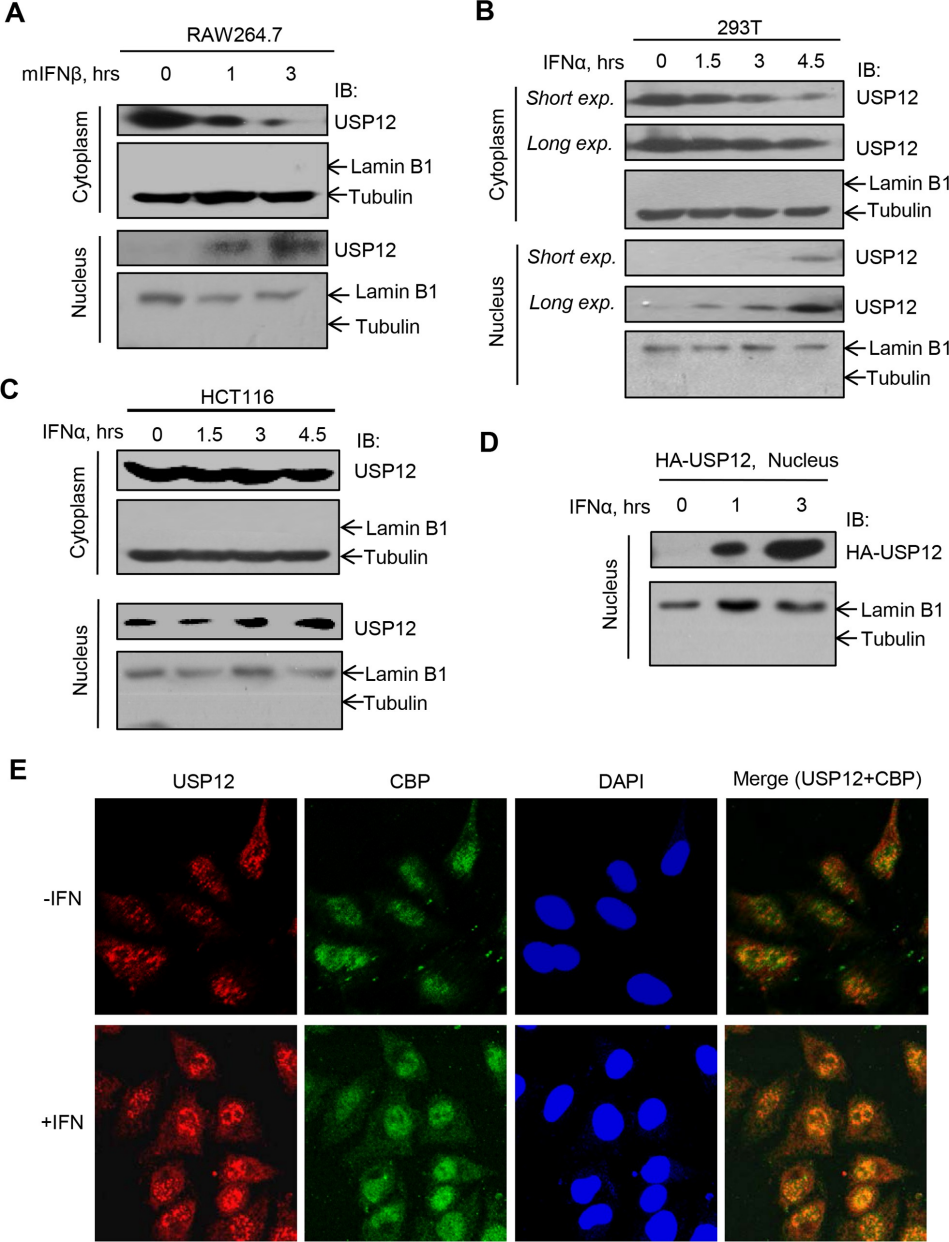
USP12 translocates from cytoplasm to nucleus in IFN-I signaling. (A) Western blot analysis of USP12 protein levels in the cytoplasm and nucleus of RAW264.7 cells treated with mouse mIFNβ (500 IU/ml) for 0, 1 and 3 hrs. (B and C) Western blot analysis of USP12 protein levels in the cytoplasm and nucleus of HEK293T (B) or HCT116 (C) cells treated with IFNα (1,000 IU/ml) for 0, 1.5, 3 and 4.5 hrs. (D) Western blot analysis of HA-USP12 levels in the nucleus of HEK293T cells transfected with HA-USP12 and then treated with IFNα (1,000 IU/ml) as indicated. (E) HeLa cells were treated with IFNα (3,000 IU/ml) for 6 hrs. Cellular CBP and USP12 proteins were stained by specific antibodies, and cell nuclei were stained by DAPI. The fluorescent images were captured with the Nikon A1 confocal microscope.

Given that the USP12/CBP interaction blocks IFN signaling activation in the cytoplasm and that CBP translocates from the nucleus to the cytoplasm, we next wanted to determine whether and how USP12 translocates between the nucleus and cytoplasm during IFN signaling. It has been reported that in TCR signaling, USP12 translocates from the nucleus to the cytoplasm to deubiquitinate LAT and Trat1 proteins [27]. Here, we treated RAW264.7 cells with mouse IFNβ (mIFNβ) for different times. To our surprise, we observed that during mIFNβ treatment, cytoplasmic USP12 protein levels dramatically decreased and nuclear USP12 protein levels increased accordingly (Fig 4A), suggesting that USP12 translocates from the cytoplasm to the nucleus in IFN signaling. This translocation manner is totally different from that in TCR signaling. To confirm this dynamic regulation of USP12, we used two additional cell lines, HEK293T and HCT116 cells. Consistently, IFNα treatment induced USP12 nuclear translocation in both HEK293T (Fig 4B) and HCT116 (Fig 4C) cells. This phenomenon was further confirmed by the nuclear import of exogenously expressed HA-USP12 in IFN signaling (Fig 4D). To directly observe the nuclear translocation of USP12, we used HeLa cells to perform an immunofluorescence assay by confocal microscopy. The results showed that endogenous USP12 interacted with endogenous CBP in intact cells, and IFNα treatment promoted USP12 nuclear accumulation (Fig 4E and S3 Fig). Collectively, we demonstrated that IFN signaling strongly stimulates USP12 translocation from the cytoplasm to the nucleus.

### USP12 maintains nuclear p-STAT1 levels by blocking acetylation and subsequent dephosphorylation of p-STAT1 in the nucleus

CBP has been reported to acetylate p-STAT1 in the nucleus, which results in the binding of the phosphatase TCPTP to p-STAT1, and subsequent dephosphorylation and downregulation of p-STAT1. Similarly, we also found that mutation of two reported key acetylation sites, Lys410 and Lys413, resulted in increased levels of p-STAT1 in cells after 2 hours of IFN treatment (S4A Fig), suggesting that STAT1 acetylation at Lys410 and Lys413 could not significantly affect STAT1 phosphorylation and activation at the initial stage of IFN treatment, but regulate p-STAT1 levels in the nucleus as a negative feedback signal, which is consistent with CBP-mediated regulation of p-STAT1 acetylation and levels in the nucleus at the late stage of IFN treatment.

**Fig 5:**
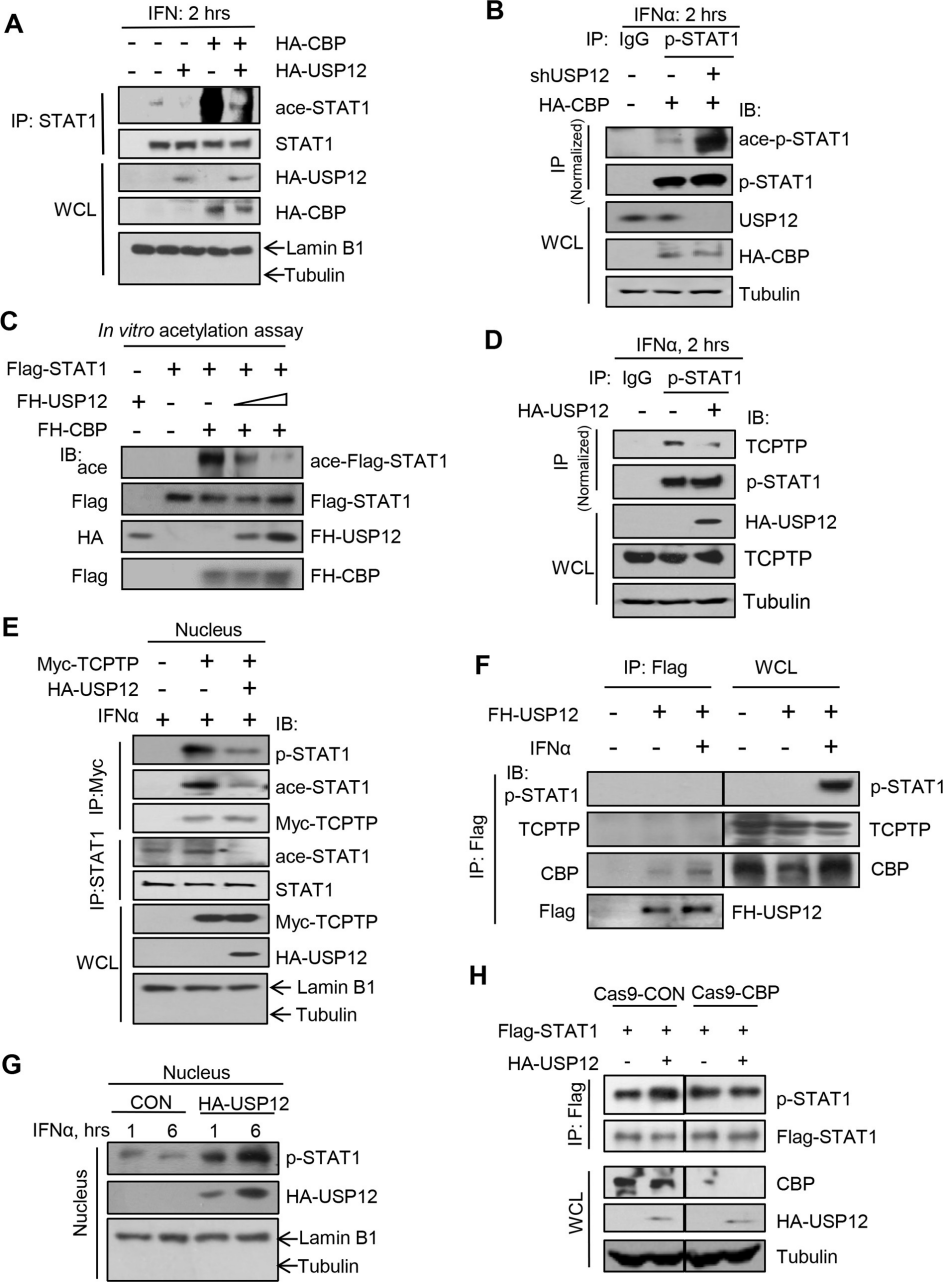
USP12 maintains nuclear p-STAT1 levels by blocking acetylation and subsequent dephosphorylation of p-STAT1 in the nucleus. (A) Immunoprecipitation analysis of STAT1 acetylation in the nucleus of RAW264.7 cells cotransfected with HA-CBP and (or) FH-USP12 as indicated, and then treated with mouse IFNβ (500 IU/ml) for 2 hrs. (B) Immunoprecipitation analysis of pY701-STAT1 (p-STAT1) acetylation in HEK293T cells cotransfected with HA-CBP and (or) shUSP12 as indicated, and then treated with IFNα (1,000 IU/ml) for 2 hrs. (C) *In vitro* acetylation assay for analysis of the effects of FH-USP12 on FH-CBP-mediated Flag-STAT1 acetylation. (D) Immunoprecipitation analysis of the interaction between p-STAT1 and TCPTP in HEK293T cells transfected with HA-USP12 and then treated with IFNα (1,000 IU/ml) for 2 hrs. (E) Immunoprecipitation analysis of the interaction between p-STAT1, ace-STAT1 and TCPTP in the nucleus of HEK293T cells transfected with Myc-TCPTP and HA-USP12, followed by IFNα (1,000 IU/ml) treatment for 2 hrs. (F) Immunoprecipitation analysis of the interaction between FH-USP12 and p-STAT1 or TCPTP or CBP in HEK293T cells transfected with FH-USP12 and then stimulated with IFNα (1,000 IU/ml) as indicated. (G) Western blot analysis of p-STAT1 levels in the nucleus of HEK293T cells transfected with or without HA-USP12 and then treated with IFNα (1,000 IU/ml) as indicated. (H) Immunoprecipitation analysis of tyrosine 701 phosphorylation of Flag-STAT1 in CBP^+/+^ (Cas9-CON) or CBP^-/-^ (Cas9-CBP) HEK293T cells cotransfected with Flag-STAT1, together with either vectors or HA-USP12.

Given that we have demonstrated that USP12 translocates into the nucleus in IFN signaling, we next sought to determine whether USP12 regulates p-STAT1 levels through CBP. Firstly, we noticed that HA-CBP interacted with Flag-STAT1 and induced Flag-STAT1 acetylation (Fig 2E, lane 3). Overexpression of USP12 did not noticeably affect the interaction between HA-CBP and Flag-STAT1 but significantly blocked Flag-STAT1 acetylation (Fig 2E, lane 3 vs. 4), suggesting that USP12 could inhibit CBP-mediated acetylation of STAT1. Furthermore, in IFN signaling, CBP-mediated acetylation of STAT1 was inhibited by USP12 overexpression (Fig 5A). Knockdown of USP12 promoted CBP-mediated acetylation of p-STAT1 induced by IFN (Fig 5B). We next performed an *in vitro* acetylation assay to observe the role of USP12 in CBP-mediated STAT1 acetylation. The results showed that CBP induced Flag-STAT1 acetylation *in vitro*, which was gradually inhibited by increasing amounts of FH-USP12 (Fig 5C), suggesting that USP12 blocks the acetylation effect of CBP on STAT1.

Given that USP12 inhibits p-STAT1 acetylation, we next observed the binding of TCPTP with p-STAT1. We found that overexpression of USP12 restricted the interaction between IFN-induced p-STAT1 and endogenous TCPTP in HEK293T cells (Fig 5D) and HeLa cells (S4B Fig). The inhibitory effect of USP12 on the p-STAT1 and TCPTP interaction was further confirmed in the nucleus (Fig 5E). Consistently, the IFN-induced interaction between p-STAT1 and TCPTP was inhibited by USP12 overexpression (S4C Fig). However, USP12 did not inhibit the interaction between p-STAT1 and another phosphatase, SHP2 (S4D Fig). Moreover, we demonstrated that in IFN signaling, USP12 interacted with CBP but not with either p-STAT1 or TCPTP (Fig 5F), suggesting that USP12 affects the p-STAT1 and TCPTP interaction through targeting CBP. In line with the decreased binding of TCPTP, IFN-induced p-STAT1 levels in the nucleus were substantially enhanced by USP12 overexpression (Fig 5G). Importantly, knockout of CBP abolished USP12-mediated upregulation of p-STAT1 levels (Fig 5H). Taken together, these findings suggest that USP12 maintains nuclear p-STAT1 levels by blocking CBP-mediated acetylation and subsequent TCPTP-mediated dephosphorylation of p-STAT1 in the nucleus.

### USP12 sustains cellular p-STAT1 levels and promotes IFN signaling and antiviral response

**Fig 6:**
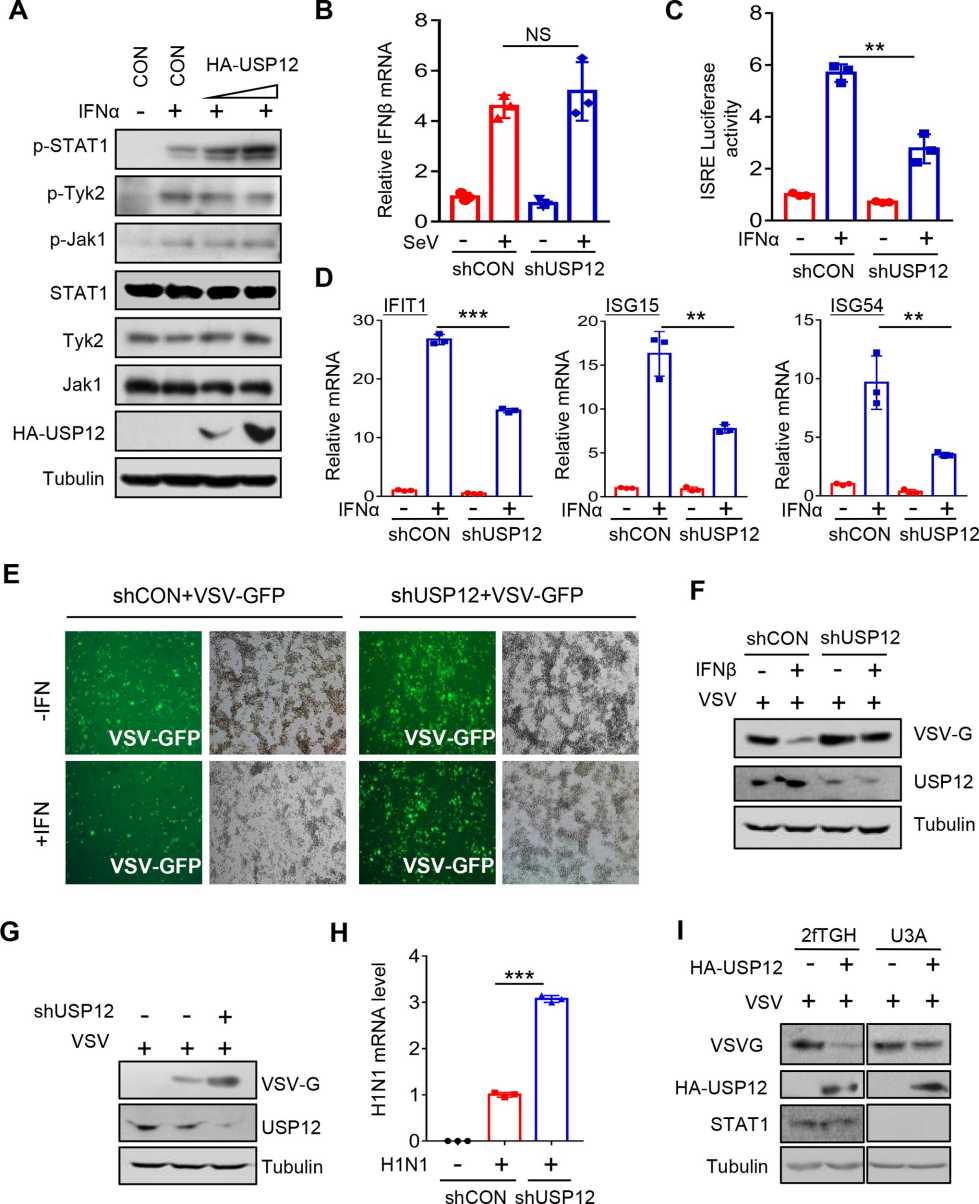
USP12 sustains cellular p-STAT1 levels and promotes IFN-I signaling and antiviral activity. (A) Western blot analysis of the levels of p-STAT1, p-Tyk2 and p-Jak1 in HEK293T cells transfected with increasing amounts of HA-USP12 and then stimulated with IFNα (1,000 IU/ml) for 3 hrs. (B) Quantitative real-time PCR (Q-PCR) analysis of IFNβ mRNA levels in RAW264.7 cells transfected with either control shRNAs (shCON) or shRNAs against USP12 (shUSP12), and then infected with SeV (MOI = 1.0) as indicated. (C) HEK293T cells were transfected with either control shRNAs (shCON) or shRNAs against USP12 (shUSP12), together with ISRE-Luc and Renilla. The luciferase activity was measured 20 hrs after IFNα (1,000 IU/ml) treatment. (D) Q-PCR analysis of representative ISGs (IFIT1, ISG15 and ISG54) mRNA levels in HEK293T cells transfected with either shCON or shUSP12, and then treated with IFNα (1,000 IU/ml) for 8 hrs. (E) RAW264.7 cells transfected with control shRNAs (shCON) or shRNAs against USP12 (shUSP12) were treated with mouse IFNβ (30 IU/ml) overnight. Cells were challenged by VSV-GFP (MOI = 0.5). After 24 hrs, VSV-GFP levels were analyzed by fluorescence. (F) RAW264.7 cells transfected with shCON or shUSP12 were treated with mouse IFNβ (30 IU/ml) overnight, and then cells were challenged by VSV (MOI = 1.0). After 20 hrs, the levels of VSV-G, USP12 and Tubulin were immunoblotted as indicated. (G) RAW264.7 cells were transfected with shCON or shUSP12, and then cells were challenged by VSV (MOI = 1.0). After 20 hrs, the levels of VSV-G, USP12 and Tubulin were analyzed using indicated antibodies. (H) RAW264.7 cells transfected with shCON or shUSP12 were challenged with H1N1 (MOI = 0.2, 20 hrs). Viral RNA levels were analyzed by Q-PCR. (I) Western blot analysis of VSV-G protein levels in 2fTGH cells and U3A cells (STAT1 deficiency) transfected with HA-USP12 and then challenged with VSV (MOI = 0.5) for 20 hrs. NS, not significant (*p*>0.05). **p<0.01 and ***p<0.001 (two-tailed unpaired Student’s *t*-test). Data are shown as mean and s.d. of three biological replicates (B, C, D, H).

Given that IFN signaling strongly stimulates nuclear import of cytoplasmic USP12 (Fig 4), we speculated that in cells with USP12 overexpression, IFN signaling will deliver the majority of cytoplasmic USP12 into the nucleus (Fig 4D), which releases CBP activity for stronger activation of IFN signaling and sustains nuclear p-STAT1 levels. Thus, we speculated that USP12 overexpression could positively regulate cellular p-STAT1 levels and IFN antiviral activity. As expected, USP12 overexpression upregulated IFN-induced levels of p-STAT1 (Fig 6A and S5A Fig). However, USP46 did not upregulate p-STAT1 levels in IFN signaling (S5B Fig). We noticed that USP12 did not noticeably affect the levels of p-Tyk2 and p-JAK1 (Fig 6A). Moreover, USP12 did not promote IFNα/β production (Fig 6B) or the promoter activity of IFNβ (S6A Fig). These results suggest that USP12 could target those signaling proteins downstream of JAK1/Tyk2, which is consistent with our above observation showing that USP12 regulates p-STAT1 levels by blocking CBP’s acetyltransferase activity. ISRE-luciferase has been widely used to measure the magnitude of IFN signaling. We found that knockdown of USP12 significantly inhibited IFNα-induced ISRE-luciferase activity (Fig 6C), whereas overexpression of USP12 enhanced ISRE-luciferase activity induced by IFNα (S6B Fig). To analyze IFN signaling-induced downstream gene expression, the mRNA levels of three representative ISGs (IFIT1, ISG15, and ISG54) were detected. USP12 knockdown decreased the mRNA levels of all three ISGs induced by IFNα (Fig 6D). Conversely, USP12 overexpression markedly promoted IFNα-induced ISG mRNA expression (S6C Fig). These findings demonstrate that USP12 is a positive regulator of IFN signaling.

Next, we sought to determine whether USP12 regulates IFN-mediated antiviral activity. Vesicular stomatitis virus (VSV) has been widely used as a sensitive viral model to assess IFN antiviral activity. Using VSV-GFP, which is a VSV virus with a GFP gene, we found that knockdown of USP12 significantly inhibited IFNα-mediated antiviral activity (Fig 6E), and overexpression of USP12 promoted IFN-mediated antiviral activity (S6D and S6E Fig). Using Western blot analysis, we confirmed that USP12 enhanced IFN-mediated antiviral response (Fig 6F). Given that viral infection stimulates IFN secretion, we speculated that virus-induced IFN signaling could be enhanced by USP12. Therefore, USP12 overexpression could enhance cellular antiviral activity. In line with this speculation, knockdown of USP12 remarkably promoted VSV infection (Fig 6G), while USP12 overexpression inhibited VSV infection (S6F Fig). Similarly, USP12 knockdown promoted infection of another RNA virus H1N1 (Fig 6H). Importantly, in STAT1-deficient cells (U3A), the ability of USP12 to inhibit viral infection was largely attenuated (Fig 6I). Taken together, these findings suggest that USP12 enhances p-STAT1 signaling and IFN antiviral efficacy.

### The deubiquitinase activity of USP12 is not required for the regulation of CBP-mediated acetylation, p-STAT1 levels and IFN antiviral activity

**Fig 7:**
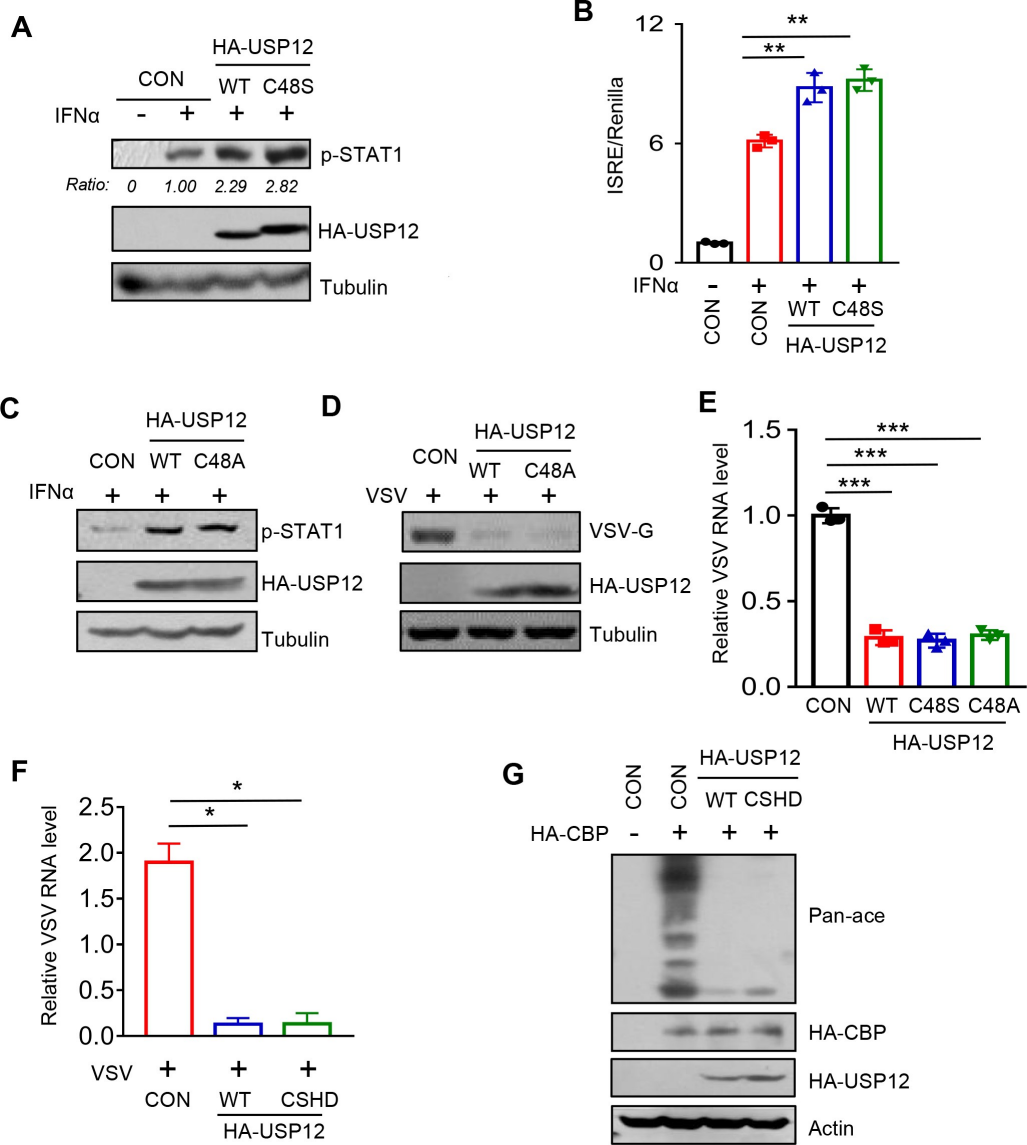
The deubiquitinase activity of USP12 is not required for the regulation of CBP-mediated acetylation, p-STAT1 levels and IFN antiviral response. (A) Western blot analysis of p-STAT1 levels in HEK293T cells transfected with HA-USP12 (WT or C48S mutant) and then stimulated with IFNα (1,000 IU/ml) for 6 hrs. (B) HEK293T cells were transfected with HA-USP12 (WT or C48S mutant), together with ISRE-Luc and Renilla. The luciferase activity was measured after IFNα (1,000 IU/ml) treatment for 20 hrs. (C) Western blot analysis of p-STAT1 levels in HEK293T cells transfected with HA-USP12 (WT or C48A mutant) and then stimulated with IFNα (1,000 IU/ml) for 6 hrs. (D) Western blot analysis of VSV-G protein levels in HEK293T cells transfected with HA-USP12 (WT or C48A mutant) and then challenged with VSV (MOI = 0.5) for 20 hrs. (E) Q-PCR analysis of VSV viral RNA levels in HEK293T cells transfected with empty vectors or HA-USP12 (WT or C48S or C48A mutant) and then challenged with VSV as (D). (F) Q-PCR analysis of VSV RNA levels in HEK293T cells transfected with HA-USP12-WT or -C48S/H173D (CSHD) and then challenged with VSV as (D). (G) Western blot analysis of pan-acetylation levels in HEK293T cells cotransfected with HA-CBP and (or) HA-USP12 (WT or C48S/H173D). *p<0.05, **p<0.01 and ***p<0.001 (two-tailed unpaired Student’s *t*-test). Data are shown as mean and s.d. of three biological replicates (B, E, F).

The aforementioned results demonstrated that USP12 binds to the HAT domain of CBP and blocks CBP’s acetylation action. In conjugation with the result showing that USP12 did not upregulate CBP protein levels (Fig 1E), we speculated that the deubiquitinase activity of USP12 could not be essential for USP12-mediated regulation of CBP action and subsequent IFN antiviral signaling. Thus, we mutated the cysteine 48 residue of USP12 to produce catalytically inactive mutants, USP12-C48S [29] and USP12-C48A [30]. We found that USP12-C48S still enhanced IFNα-induced p-STAT1 levels (Fig 7A) and promoted ISRE-luciferase activity induced by IFNα (Fig 7B). Similarly, USP12-C48A had the same ability to enhance p-STAT1 levels in IFN signaling as USP12-wild type (WT) (Fig 7C). As a consequence, both USP12-C48A (Fig 7D and 7E) and USP12-C48S (Fig 7E) significantly inhibited viral infection with a similar efficiency as USP12-WT. It has recently been reported that the histidine 173 residue (H173) is also important for the deubiquitinase activity of USP12. Thus, we further constructed a USP12-C48S/H173D (CSHD) mutant. Consistently, as compared with USP12-WT, the USP12-C48S/H173D mutant had a similar ability to inhibit viral infection (Fig 7F). Given that we have demonstrated that the effects of USP12 on IFN antiviral signaling were achieved via inhibiting CBP’s acetylation action, we further determined whether the catalytically inactive mutant of USP12 could inhibit the acetylation modification induced by CBP. Our results showed that, compared with USP12-WT, the USP12-C48S/H173D mutant can similarly inhibit CBP-mediated pan-acetylation in cells (Fig 7G). Collectively, these findings suggest that USP12 inhibits CBP-mediated acetylation and promotes IFN antiviral activity independently of its deubiquitinase activity.

### USP12 deficiency attenuates host antiviral response and IFN antiviral efficacy

**Fig 8:**
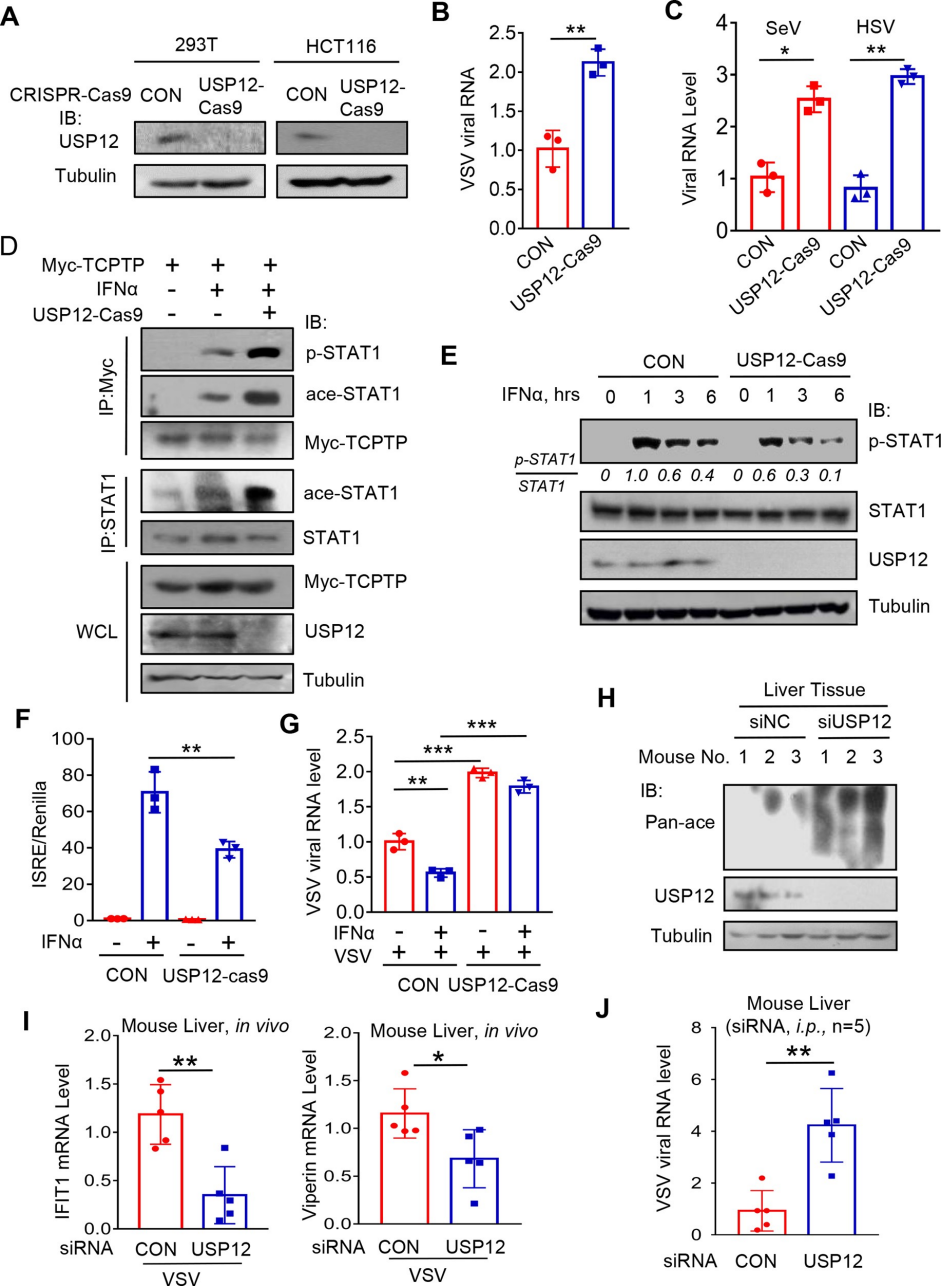
USP12 deficiency attenuates IFN signaling and antiviral activity. (A) Western blot analysis of endogenous USP12 levels in USP12^+/+^(CON) or USP12^-/-^(USP12-Cas9) HEK293T cells or HCT116 cells made by the CRISPR-Cas9-mediated genome editing. (B) USP12^+/+^(CON) or USP12^-/-^(USP12-Cas9) HEK293T cells were infected with VSV (MOI = 0.5) for 20 hrs. Viral RNA levels were analyzed by Q-PCR. (C) USP12^+/+^(CON) or USP12^-/-^(USP12-Cas9) HCT116 cells were challenged with SeV (MOI = 0.5, 20 hrs) or HSV (MOI = 1.0, 8 hrs). Viral RNA levels were analyzed by Q-PCR. (D) Immunoprecipitation analysis of the interaction between p-STAT1, ace-STAT1 and Myc-TCPTP in USP12^+/+^(CON) or USP12^-/-^(USP12-Cas9) HEK293T cells transfected with Myc-TCPTP and then treatment with IFNα (1,000 IU/ml) for 2 hrs. (E) Western blot analysis of p-STAT1 levels in USP12^+/+^(CON) or USP12^-/-^(USP12-Cas9) HEK293T cells stimulated with IFNα (1,000 IU/ml) for 0, 1, 3, 6 hrs. (F) USP12^+/+^(CON) or USP12^-/-^(USP12-Cas9) HEK293T cells were transfected with ISRE-Luc and Renilla. The luciferase activity was measured 20 hrs after IFNα (1,000 IU/ml) treatment. (G) Q-PCR analysis of viral RNA levels in USP12^+/+^(CON) or USP12^-/-^(USP12-Cas9) HEK293T cells pretreated with IFNα (30 IU/ml) overnight and then challenged by VSV (MOI = 1.0) for 20 hrs. (H) Mice (N = 3, per group) were administrated with either negative control siRNAs (siNC) or siRNAs against mouse USP12 (siUSP12) by tail vein injection. Western blot analysis of USP12 protein levels and the pan-acetylation levels in mouse livers as indicted. (I and J) Mice (N = 5, per group) were injected with siNC or siUSP12 twice (once per day). 48 hrs after initial injection, mice were infected with VSV (2X10^8^ pfu per mouse) for 24 hrs. The representative ISGs (IFIT1, Viperin) mRNA (I) or Viral RNA (J) levels in mouse liver tissues were analyzed by Q-PCR. *p<0.05, **p<0.01 and ***p<0.001 (two-tailed unpaired Student’s *t*-test). Data are shown as mean and s.d. of three biological replicates (B, C, F, G, I, J).

To further understand the physiological function of USP12 in cells, we knocked out cellular USP12 by CRISPR-Cas9-mediated genome editing in both 293T and HCT116 cells (Fig 8A). We observed that USP12 deficiency largely attenuates cellular antiviral ability against VSV (Fig 8B). Consistently, the results from experiments with other viruses, including SeV and HSV, supported that USP12 is a positive regulator of cellular antiviral ability (Fig 8C). Mechanistically, USP12 deficiency promoted the binding of TCPTP to p-STAT1 (Fig 8D), thus decreasing p-STAT1 levels in IFN signaling (Fig 8E). As a consequence, USP12 deficiency significantly inhibited IFN-induced ISRE-luciferase activity (Fig 8F). Importantly, in USP12-deficient cells, IFN-mediated antiviral efficacy was dramatically inhibited (Fig 8G).

Moreover, we utilized an *in vivo* siRNA strategy to knock down mouse USP12. We found that the levels of USP12 in mouse livers significantly decreased by the injection of siRNAs against USP12 (siUSP12) (Fig 8H). Consistently, the pan-acetylation levels of liver tissues in the siUSP12 mice were higher than that in control siRNAs (siNC) mice (Fig 8H). When these mice were challenged with VSV viruses, cellular ISGs levels in mouse livers were significantly attenuated by siUSP12 injection (Fig 8I). As a consequence, the viral loads were higher in the livers of mice administered siUSP12 than those of mice administered control siRNAs (Fig 8J). These findings suggest that USP12 critically regulates host IFN antiviral response *in vivo*.

**Fig 9:**
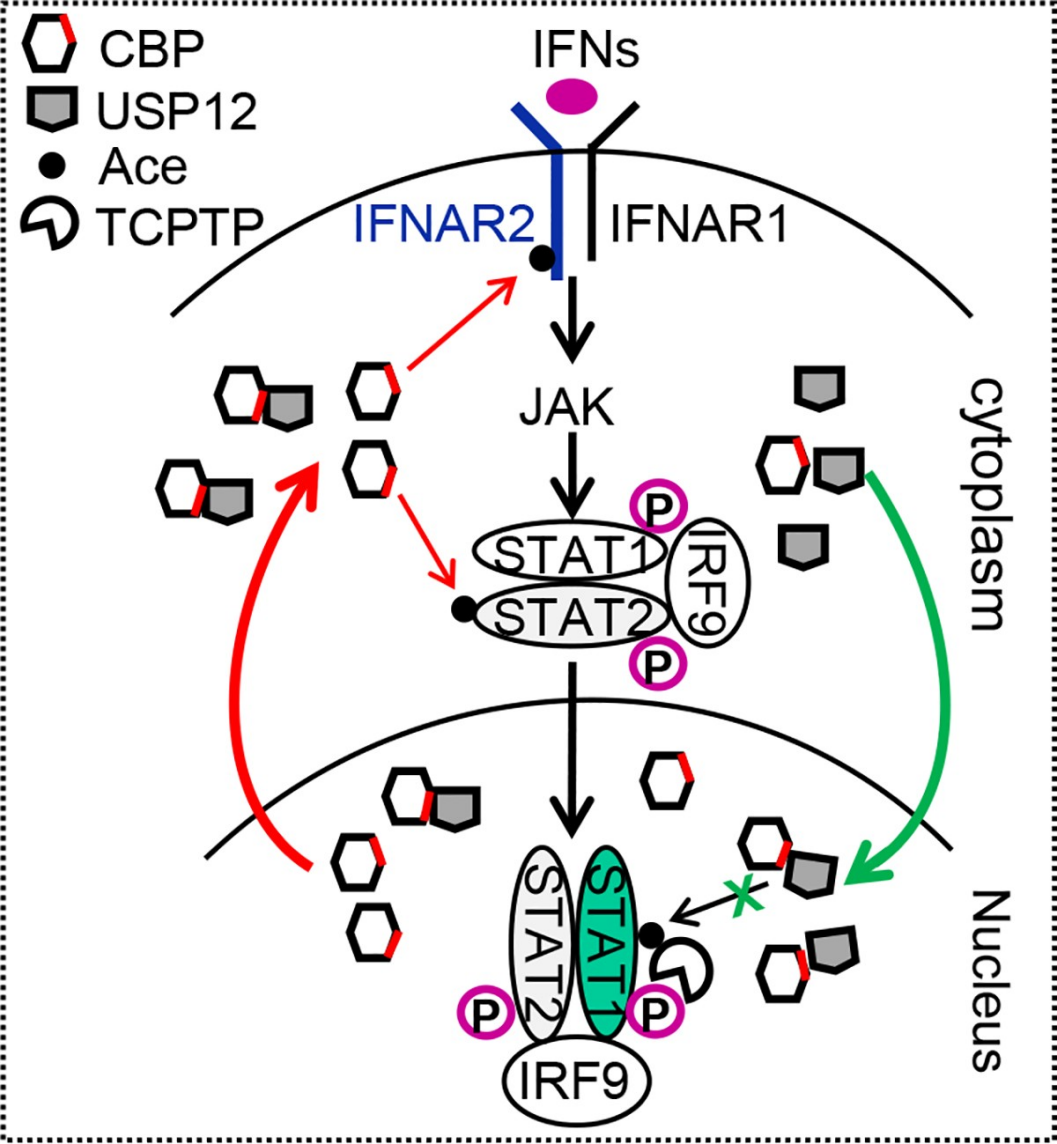
A proposed model of regulation of the IFN-JAK/STAT1 signaling by USP12. USP12 translocates from cytoplasm to nucleus in IFN signaling. The decrease in cytoplasmic USP12 levels facilitates CBP-mediated acetylation and activation of IFN signaling. USP12 accumulation in the nucleus maintains activated p-STAT1 levels by blocking CBP-mediated acetylation of p-STAT1 and subsequent TCPTP-mediated dephosphorylation of p-STAT1, which enables efficient IFN antiviral response.

Our study showed that IFN-induced p-STAT1 signaling undergoes delicate regulation in the nucleus. A large number of CBP proteins in the nucleus severely endanger nuclear p-STAT1 levels by inducing TCPTP-mediated dephosphorylation of p-STAT1. However, IFN signaling stimulates the nuclear import of USP12, which blocks CBP’s acetylation action by binding with its HAT domain in the nucleus, thus playing pivotal roles in maintaining nuclear p-STAT1 levels and efficient IFN antiviral activity (Fig 9). In addition, USP12 translocation from the cytoplasm to the nucleus facilitates CBP-induced acetylation and activation of IFN signaling proteins in the cytoplasm (Fig 9). Taken together, our study reveals that USP12 is a critical regulator of IFN antiviral response.

## Discussion

The acetyltransferase CBP plays critical roles in transcription regulation by acetylating a multitude of cellular proteins, including signaling proteins and transcription-related factors. In light of its significance in essential cellular activities, studies on the regulation of CBP’s acetyltransferase activity have been attracting great interest. Some PTMs of CBP that are involved in its acetyltransferase activity have been reported [22, 23, 31–33]. In addition, viral proteins such as the adenovirus EIA, E6 and E7 proteins of HPV, and the Tax protein of HTLV [34–36] can also affect the acetyltransferase activity of CBP. However, cell-intrinsic inhibitors of CBP have not been identified. In this study, we demonstrated that USP12 can bind with the HAT domain of CBP *in vivo* and *in vitro*. As a result, USP12 inhibits CBP-induced acetylation of cellular proteins. These findings reveal an intrinsic inhibitory regulation of CBP’s acetyltransferase activity.

USP12, as one member of the deubiquitinases, regulates the stability and levels of several cellular proteins dependently on its deubiquitinase activity [25–27]. However, our study clearly uncovers that USP12 affects CBP action independently of its deubiquitinase activity, which is not surprising. Given that USP12 binds to the HAT domain of CBP and blocks its acetyltransferase domain, the deubiquitinase activity of USP12 is not required for its inhibitory effect on CBP function. We also demonstrated that USP12 interacted with CBP, but not either p-STAT1 or TCPTP, suggesting that the regulation of USP12 on both TCPTP binding and p-STAT1 levels is through CBP. Thus, our study uncovers a noncatalytic regulatory mechanism of USP12 in innate antiviral immunity.

CBP has both a stimulatory and inhibitory effect on many of cellular signaling pathways, depending on different protein substrates targeted by CBP. This makes it difficult to observe the final effects of CBP activity deficiency on these signaling pathways. In IFN signaling, CBP was reported to translocate from the nucleus to cytoplasm, which plays essential roles in acetylation and activation of IFN signaling proteins and the formation of the ISGF3 signaling complex [28]. Thus, we turned to IFN signaling to study the significance of the CBP/USP12 interaction. To our surprise, in IFN signaling USP12 translocates from the cytoplasm to the nucleus. Furthermore, we demonstrated that the decrease in USP12 in the cytoplasm markedly promoted CBP-induced acetylation of IFNAR2 and STAT2, which resulted in increased formation of the ISGF3 complex. Thus, USP12 translocation to the nucleus facilitates IFN signaling activation in the cytoplasm. Moreover, we further found that the increase in nuclear USP12 blocked CBP-mediated acetylation of p-STAT1, thus inhibiting the binding of TCPTP and sustaining nuclear p-STAT1 levels and subsequent IFN antiviral signaling. Our findings reveal an inverse translocation of USP12 and CBP as an efficient regulatory mechanism for IFN signaling activation and maintenance.

A recent report showed that in TCR signaling, USP12 translocates from the nucleus to the cytoplasm, where USP12 deubiquitinates and stabilizes LAT and Trat1 and prevents their lysosome-dependent degradation in T cells. This report about USP12 translocation in TCR signaling is different from our findings in IFN signaling. We demonstrated that in many different types of cells, IFN signaling can stimulate the nuclear import of USP12. The different translocation manners of USP12 in various signaling pathways suggest that USP12 undergoes dynamic regulation in different cellular compartments for efficient signaling delivery or activation.

This study identifies USP12 as an intrinsic inhibitor of CBP in cells. The USP12/CBP interaction may provide a critical balance for cellular acetylation regulation, since CBP possesses such broad-spectrum and potent acetylation abilities. Breaking this balance could push the activation of cellular signaling pathways induced by extracellular or intracellular stimulants (Fig 9). As demonstrated in our study, IFN signaling stimulates USP12 translocation from the cytoplasm to the nucleus, which maintains IFN-induced p-STAT1 levels in the nucleus for efficient antiviral activity. Uncovering this USP12/CBP interaction and its significance in signaling pathways not only extends our understanding of the essential regulation of CBP acetyltransferase activity but also promotes the development of new strategies for the intervention of disease-related signaling pathways.

## Materials and methods

### Mice

C57BL/6 mice were purchased from the Laboratory Animal Center of Soochow University. Mice were maintained and bred in special-pathogen-free (SPF) conditions in the Experimental Animal Center of Soochow University. 6–8 weeks old mice were used in most of experiments.

### Ethics statement

Animal care and use protocol adhered to the National Regulations for the Administration of Affairs Concerning Experimental Animals. All protocols and procedures for mice study were approved by the ethics committee of the Scientific Investigation Board of Soochow University (Identification No. 201901A166), and were performed in accordance with the Laboratory Animal Management Regulations of the Scientific Investigation Board of Soochow University.

### Cells isolation from mice

Mouse tissues were prepared from the 6–8 weeks adult mice. Briefly, mouse tissues were cut into pieces and grinded to cell suspension. Mouse primary lung cells and liver cells were collected and prepared for further experiments. Bone marrows were derived from 8 weeks old mice, and the cells were cultured in RPMI medium and then stimulated by GM-CSF (50 ng/ml) for BMDMs differentiation.

### Cell Lines, plasmids, transfection and reagents

HEK293T (human embryonic kidney 293T), HCT116 (human colon cancer), A549 (lung adenocarcinoma), HeLa (human cervical cancer) and RAW264.7 (mouse peritoneal macrophage) cells were obtained from American Type Culture Collection. 2fTGH (human fibrosarcoma) cells were gifts from Dr. S.Y. Fuchs (University of Pennsylvania). U3A (STAT1-deficient human fibrosarcoma) cells were obtained from Dr. GQ Chen. Cells were cultured in Dulbecco’s modified Eagle’s medium (DMEM; HyClone) supplemented with 10% FBS (GIBCO, Life Technologies), 1% penicillin-streptomycin at 37°C under 5% CO_2_.

HA-USP12, USP12-C48S and shUSP12 were nice gifts from Dr. Christel Brou (Institut Pasteur, France). USP12-C48A and USP12-C48S/H173D were generated by QuickChange Lightning site-Directed Mutagenesis Kit (Stratagene). Flag-HA-USP12 was generated in the pOZ-FH vector (Addgene #32516) using PCR amplification from HA-USP12. HA-USP46 was kindly provided by Dr. J. Wade Harper (Harvard Medical School, Addgene plasmids). ISRE-Luc, Flag-STAT1, IFNβ-Luc, and Renilla plasmids were described as previously. Bacterially expressed His-CBP (989–1317) plasmids were a nice gift from Dr. Peter E. Wright (the Scripps Research Institute), and bacterially expressed His-CBP (1324–1700) plasmids were purchased from Addgene (#99340). Bacterially expressed GST-USP12 plasmids were generated by PCR amplification from the cDNA of RAW264.7 cells. HA-CBP, Flag-HA-CBP and HA-p300 were nice gifts from Dr. T. Kang (Sun Yat-sen University, China). TCPTP and Myc-SHP2 were gifts from Dr. Y. E. Chinn (Soochow University, China). Myc-His-TCPTP was generated by PCR from TCPTP plasmids. All the plasmids were confirmed by DNA sequencing. All transient transfections were carried out using LongTrans (UCallM, TF/07) according to the manufacturer’s instruction. Recombinant human IFNα was purchased from PBL Interferon Source. IFNα was used at the concentration of 1,000 IU/ml, unless stated otherwise.

### Immunoprecipitation and immunoblotting

For immunoblotting, cells were harvested using Nonidet P-40% lysis buffer containing 150 mM NaCl, 20 mM Tris-HCl (PH 7.4), 1% Nonidet P-40, 0.5 mM EDTA, 0.2 mM Na3VO4, 0.5 mM NaF and PMSF (50 μg/ml). When protein ubiquitination was examined, RIPA buffer (Beyotime) was used and N-ethylmaleimide (10 mM) was added into the lysis buffer. The collected supernatant was incubated with specific antibodies overnight at 4°C on a rotor. Next, Protein G agarose beads (Millipore, #16–266) were added and incubated for additional 3 hrs on a rotor at 4°C. After washing three times with high-salt (500 mM NaCl) washing buffer and twice with normal washing buffer (150 mM NaCl, 20 mM Tris-HCl (PH 7.4), 1% Nonidet P-40, 0.5 mM EDTA), the immunoprecipitates were eluted by boiling with loading buffer containing β–mercaptoethanol for 10 min and analyzed by SDS-PAGE, followed by transferring to PVDF membranes. The membranes were probed with the primary antibodies overnight at 4°C, followed by the secondary antibodies (HRP-conjugated Goat anti-mouse or Goat anti-rabbit). SuperSignal West Dura Extended kits (Thermo Scientific) were used to visualize the immunoreactive bands. For immunoprecipitation of Flag-tagged proteins, M2 Affinity Gel (Sigma, A2220) was used in lysates for 4 hrs on a rotor at 4°C. Image J program (http://rsbweb.nih.gov/ij/download.html) was used for densitometric analysis of western blotting.

In this article, following antibodies were used: antibodies against USP12 (1:500, Abcam, #ab89870), pY701-STAT1 (1:1,000, Cell Signaling, #9167), p-JAK1 (1:200, Santa Cruz, sc-16773), p-Tyk2 (1:500, Cell Signaling, #9321), STAT1 (1:1,000, Cell Signaling, #9172), JAK1 (1:1,000, Santa Cruz, sc-1677), Tyk2 (1:500, Cell Signaling, #14193), TCPTP (1:500, Santa Cruz, sc-373835), CBP (1:1,000, Cell Signaling, #7389), p300 (1:500, Santa Cruz, sc-48343), Acetylated-Lysine (1:1,000, Cell Signaling, #9441), VSV-G (1:2,000, Santa Cruz, sc-66180), HA (1:1,000, Abcam, ab9110), Flag (1:5,000, Sigma, F7425), Myc (1:2,000, Abmart, M20002H), β-Actin (1:1,000, Proteintech, #66009-1-Ig), Lamin B1 (1:1,000, Proteintech, 12987-1-AP), Alpha-Tubulin (1:5,000, Proteintech, 66031-1-Ig). Image J program (http://rsbweb.nih.gov/ij/download.html) was used for densitometric analysis of western blots.

### Cytoplasmic and nuclear proteins extraction

Cells were washed in cold PBS and then harvested in harvest lysis buffer containing 10 mM HEPES (PH 7.9), 50 mM NaCl, 0.5 mM Sucrose, 0.1 mM EDTA, 0.5% Triton X-100, 1mM DTT, 10 mM Sodium pyrophosphate decahydrate, 0.5 M NaF, 0.2 M Na_3_VO_4_, 1 mM PMSF and protease inhibitor mixtures (Sigma) on ice. The supernatant was collected for the cytoplasmic extract after centrifuging for 5 min at 1,500 rpm. The pellet was resuspended with Buffer A containing 10 mM HEPES (PH 7.9), 10 mM KCl, 0.1 mM EGTA, 0.1 mM EDTA, 1 mM DTT, 1 mM PMSF and protease inhibitors mixtures. Centrifuge for 5 min at 1,500 rpm and then remove the supernatant. Then four volume of buffer C containing 10 mM HEPES (PH 7.9), 500 mM NaCl, 0.1 mM EGTA, 0.1 mM EDTA, 0.1% Nonidet P-40, 1 mM DTT, 1 mM PMSF and protease inhibitors mixtures were added. Vortex for 30 min at 4°C and then centrifuge for 10 min at 14,000 rpm. The supernatant was collected as the nuclear extract. Tubulin-α and Lamin B1 were used as loading controls for the cytoplasm and nucleus, respectively.

### Mass spectrometry analysis (MS)

SDS-PAGE gels were stained with the Silver Staining kits (Beyotime, P0017S). The gel bands from control and experimental groups were carefully excised, and then were digested with trypsin. The resulting tryptic peptides were purified using C18 Zip Tip. Then the peptides were analyzed by an Orbitrap Elite hybrid mass spectrometer (Thermo Fisher) coupled with a Dionex LC. The peptide spectrum matches (PSMs) for Flag-HA-USP12 were obtained after the database search using Proteome Discoverer 1.4 against a UniProt protein database containing 89,796 entries with the addition of 247 common contaminants.

### *In vitro* acetylation assay

HEK293T cells were transfected with Flag-STAT1, or Flag-HA-USP12, or Flag-HA-CBP. Cells were harvested and the lysates were subjected to immunoprecipitation with M2 beads (anti-Flag). Flag immunoprecipitates were eluted with the Flag peptides (Sigma). Then Flag-STAT1, Flag-HA-USP12 and Flag-HA-CBP were added to the acetylation reaction buffer (1 mM DTT, 20 μM Ac-CoA, 50 μM Tris-HCl, PH 8.0, 0.1 mM EDTA, 10% glycerol) for reaction at 30°C for 60 min. Flag-STAT1 was immunoprecipitated by STAT1 antibodies and the acetylation levels were analyzed using SDS-PAGE and western blotting.

### Expression of prokaryotic proteins

Total RNAs were isolated from RAW264.7 cells using TRIzol reagent (Invitrogen). The cDNA was produced by reverse transcription using oligo (dT). Mouse USP12 primers were used to construct bacterially expressed plasmids. The primers are as following: *USP12* forward, 5’-CGCGGATCCCCATGGAAATCCTAATGACAGTC-3’ and reverse, 5’-CCGCTCGAGGTCCCGAGACTGATAGAAAA-3’. The PCR products were cloned into pGEX-5X-1 vector and were expressed in E. coli. BL21.

The E. coli. BL21 bacteria containing USP12 expression plasmids were picked and put into 300 ml liquid LB medium for shaking at 37°C. When OD600 of liquid medium was 0.6–0.8, IPTG (final concentration 1 mM) was added to LB for an additional 12 h culture at 16°C. After centrifuging, the bacteria precipitation was resuspended in 1 x PBS and then was lysed. The lysates were further centrifuged, and the supernatant was collected and incubated with DNase I for 1 h at 4°C. Then the supernatant was incubated with specific antibody columns overnight at 4°C. After adding elution buffer, the bacterially expressed proteins were collected and used for further binding assay.

### Reporter gene assay

For analysis of IFN-β production, cells were transfected with IFNβ-luciferase and Renilla plasmids, together with or without HA-USP12. After 48 hrs, cells were infected with SeV for 24 hrs and then collected. For analysis of IFN-induced transcriptional activity, cells were transfected with the ISRE-Luciferase together with Renilla plasmids and (or) HA-USP12. After 48 hrs, cells were treated with IFNα for 24 hrs. Then the Luciferase activity was measured using the Dual-Luciferase Reporter Assay System (Promega, #E1910).

### RNA isolation and real-time PCR

Total RNAs were extracted from different cells using TRIzol reagent (Invitrogen). The cDNA was produced by reverse transcription using oligo (dT), and then analyzed by quantitative real-time PCR (Q-PCR) using IFIT1, ISG15, ISG54, SeV, H1N1, HSV-UL46, VSV, β-actin, IFN-α, and IFN-β specific primers and SYBR GreenSupermix (Bio-Rad Laboratories). The primer sequences were as follows:

IFIT1, 5‘-CACAAGCCATTTTCTTTGCT-3’

and 5‘-ACTTGGCTGCATATCGAAAG-3’;

ISG15, 5‘-GGGACCTGACGGTGAAGATG-3’

and 5‘-CGCCGATCTTCTGGGTGAT-3’;

ISG54, 5‘-CACCTCTGGACTGGCAATAGC-3’

and 5‘-GTCAGGATTCAGCCGAATGG-3’;

VSV, 5‘-ACGGCGTACTTCCAGATGG-3’

and 5‘-CTCGGTTCAAGATCCAGGT-3’;

SeV, 5‘- GATGACGATGCCGCAGCAGTAG-3’

and 5‘- CCTCCGATGTCAGTTGGTTCACTC-3’;

H1N1, 5‘-TTCTAACCGAGGTCGAAACG-3’

and 5‘-ACAAAGCGTCTACGCTGCAG-3’;

HSV-UL46, 5′-CTTGCCGGTCTGCCACAG-3′

and 5′-CTCCAATCGCCGGTTCCTCC-3′;

IFN-α, 5‘-TGGGAACAGAGCCTCCTAGA-3’

and 5‘-CAGGCACAAGGGCTGTATTT-3’;

IFN-β, 5‘-CATTACCTGAAGGCCAAGGA-3’

and 5‘-CAGCATCTGCTGGTTGAAGA-3’;

β-actin, 5‘-ACCAACTGGGACGACATGGAGAAA-3’

and 5‘-ATAGCACAGCCTGGATAGCAACG-3’.

The relative expression of the target genes was normalized to β-actin mRNA. The results were analyzed from three independent experiments and were shown as the average mean ±SD.

### Viral infection *in vitro*

Vesicular stomatitis virus (VSV) and Sendai virus (SeV) were obtained from Dr. Chen Wang (Shanghai Institutes for Biological Science, Chinese Academy of Science, China). Influenza A Virus (H1N1, PR/8/34) was a gift from Dr. Jianfeng Dai (Soochow University, China). Herpes simplex virus (HSV) was provided by Dr. Chunfu Zheng (Fujian Medical University, China). VSV-GFP was a gift from Dr. Chunsheng Dong (Soochow University, China). When IFN antiviral activity was determined, cells were pretreated with IFNα (50 IU/ml) for 16 hrs. After washing twice, cells were challenged by VSV or VSV-GFP at a multiplicity of infection (MOI) of 0.5 for 2 hrs. The infection medium was removed by washing twice. Cells were fed with fresh medium containing 10% FBS and incubated for another 24 hrs. Immunofluorescence or western blotting was used to analyze viral infection. To assess cellular antiviral abilities against VSV, SeV, H1N1, and HSV, cells were challenged with different viruses for 2 hrs. Then the supernatant was removed and cells were returned to the medium containing 10% FBS. After 20 hrs infection, cells were collected and viral RNAs were analyzed by quantitative real-time PCR.

### Viral infection *in vivo*

The siRNAs targeting mouse USP12 (5’- GGAAUACCUGCUACUGCAAdTdT-3’) (siUSP12) were synthesized by the Biomics Biotechnologies (China). Mice were injected with negative control siRNAs (siNC) or siUSP12 by tail vein injection at a dose of 37.5 μg/mice twice (once per day). 48 hrs after initial injection, mice were infected with VSV viruses (2X10^8^ pfu per mouse) for 24 hrs. The livers were taken to detect the levels of VSV and ISGs by quantitative real-time PCR (Q-PCR), or the levels of pan-acetylation and USP12 protein by western blotting.

### CRISPR-Cas9-mediated genome editing

Small guide RNAs targeting human USP12 (5’-CACCGATTCGCCTCCA TCTGTACCA-3’) were cloned into the lentiCRISPRv2 vector, which was a nice gift from Dr. Fangfang Zhou (Soochow University, China). The constructs were then transfected into HEK293T cells. 48 hrs after transfection, the supernatant was collected and used to infect either HEK293T or HCT116 cells. After 48 hrs, the infected cells were placed under puromycin (1.5 μg/ml) selection for one week and then were transferred to 96-well plates to grow until further experiments.

### Immunofluorescence and confocal microscopy assay

For immunofluorescence microscopy analyses, VSV-GFP-infected cells were pictured with upright fluorescence microscope (Tokyo, Japan). Magnification was 200. Immunofluorescence Confocal assay was stated as described previously. Briefly, cells were stimulated with IFNs and fixed in 4% paraformaldehyde at room temperature. Then cells were permeabilized with 0.5% Triton X-100 and blocked with 5% BSA. Cells were incubated with either an anti-USP12 antibody (1:50, Santa Cruz, sc-82072) or an anti-CBP antibody (1:50, Thermofisher, MA5-13634) overnight, followed by staining with either 488 goat anti-mouse IgG (Alexa Fluor, A11001) or 594 goat anti-rabbit IgG (Alexa Fluor, A11012). Cell nuclei were stained with DAPI for 1 min, and the fluorescent images were captured with the Nikon A1 confocal microscope.

### Statistical analysis

Two-tailed Student’s t-test was used to analyze the comparison between different groups. Data represent the mean ± sd. All differences were considered statistically significant when p < 0.05.

## Supporting information

S1 Fig USP12 interacts with CBP. (A) Immunoprecipitation analysis of the interaction between Flag-HA-USP12 (FH-USP12) and HA-CBP in HEK293T cells. (B) Immunoprecipitation analysis of the interaction between CBP and USP12 in HEK293T cells transfected with empty vectors (-) or FH-USP12. (C) Western blot analysis of HA-CBP levels in HEK293T cells cotransfected with either empty vectors (CON) or increasing amounts of HA-USP12, together with HA-CBP.
(TIF)

S2 Fig USP12 interacts with p300 and regulates p300 ubiquitination and protein levels. (A) Western blot analysis of p300 protein levels in RAW264.7 cells transfected with either control shRNAs (shCON) or shRNAs against USP12 (shUSP12). (B) Immunoprecipitation analysis of the interaction between FH-USP12 and HA-p300 in HEK293T cells. (C) HEK293T cells transfected with empty vectors (-) or HA-USP12. Cells then were treated with MG132 (10 μM) for 4 h. Immunoprecipitation (IP) and immunoblotting (IB) were performed as indicated.
(TIF)

S3 Fig USP12 translocates from cytoplasm to nucleus in IFN-I signaling. HeLa cells were treated with IFNα (3,000 IU/ml) for 0, 1, 3 and 6 hrs. Cellular USP12 proteins were stained by specific USP12 antibodies, and cell nuclei were stained by DAPI. The fluorescent images were captured with the Nikon A1 confocal microscope.
(TIF)

S4 Fig USP12 inhibits the interaction between p-STAT1 and TCPTP. (A) STAT1-deficiency fibroblast cells U3A were transfected with Flag-STAT1-WT or Flag-STAT1-K410R, K413R (KKRR), followed by treatment with IFNα (1,000 IU/ml) as indicated. Phosphorylated STAT1 at Tyr701 (p-STAT1) was analyzed using a specific antibody. (B) Immunoprecipitation analysis of the interaction between p-STAT1 and TCPTP in HeLa cells transfected with HA-USP12 and then treated with IFNα (1,000 IU/ml) for 2 hrs. (C) Immunoprecipitation analysis of the interaction between p-STAT1 and TCPTP in HEK293T cells transfected with HA-USP12 and then treated with IFNα (1,000 IU/ml) for 0, 1, and 2 hrs. (D) Immunoprecipitation analysis of the interaction between p-STAT1 and Myc-SHP2 in HEK293T cells cotransfected with HA-USP12 and (or) Myc-SHP2 and then treated with IFNα (1,000 IU/ml) for 2 hrs.
(TIF)

S5 Fig USP12 but not USP46 upregulates p-STAT1 levels in IFN-I signaling. (A) Western blot analysis of p-STAT1 levels in HEK293T cells transfected with HA-USP12 and then treated with IFNα (1,000 IU/ml) for 0, 1, 3, and 6 hrs. (B) Western blot analysis of p-STAT1 levels in HEK293T cells transfected with HA-USP46 and then treated with IFNα (1,000 IU/ml) for 0, 1, 3, and 6 hrs.
(TIF)

S6 Fig USP12 promotes IFN-I signaling and antiviral response. (A) HEK293T cells were transfected with empty vectors (CON) or HA-USP12, together with IFNβ-Luciferase and Renilla. The luciferase activity was measured 20 hrs after SeV (MOI = 0.5) infection. (B) HEK293T cells were transfected with empty vertors (CON) or HA-USP12, together with ISRE-Luc and Renilla. The luciferase activity was measured 20 hrs after IFNα (1,000 IU/ml) treatment. (C) Q-PCR analysis of representative ISGs (IFIT1, ISG15 and ISG54) mRNA levels in HEK293T cells transfected with empty vectors (CON) or HA-USP12, and then treated with IFNα (1,000 IU/ml) for 8 hrs. (D) HEK293T cells transfected with empty vectors (CON) or HA-USP12 were treated with IFNα (50 IU/ml) overnight. Cells were challenged by VSV-GFP (MOI = 0.5). After 24 hrs, VSV-GFP levels were detected by fluorescence. (E) 2fTGH cells transfected with or without HA-USP12 were treated with IFNα (30 IU/ml) overnight, and then cells were challenged by VSV (MOI = 1.0). After 20 hrs, the level of VSV viral RNA was analyzed by Q-PCR. (F) Western blot analysis of VSV-G protein levels in HCT116 cells transfected with HA-USP12 and then challenged with VSV (MOI = 0.5) for 20 hrs. NS, not significant (*p*>0.05), *p<0.05 and ***p<0.001 (two-tailed unpaired Student’s *t*-test). Data are shown as mean and s.d. of three biological replicates.
(TIF)
