# Supplementary figures and images for: USP12 translocation maintains interferon antiviral efficacy by inhibiting CBP acetyltransferase activity
Source: PLoS Pathog. 2020 Jan 3;16(1):e1008215. doi: 10.1371/journal.ppat.1008215 (PMC6961928; doi:10.1371/journal.ppat.1008215)

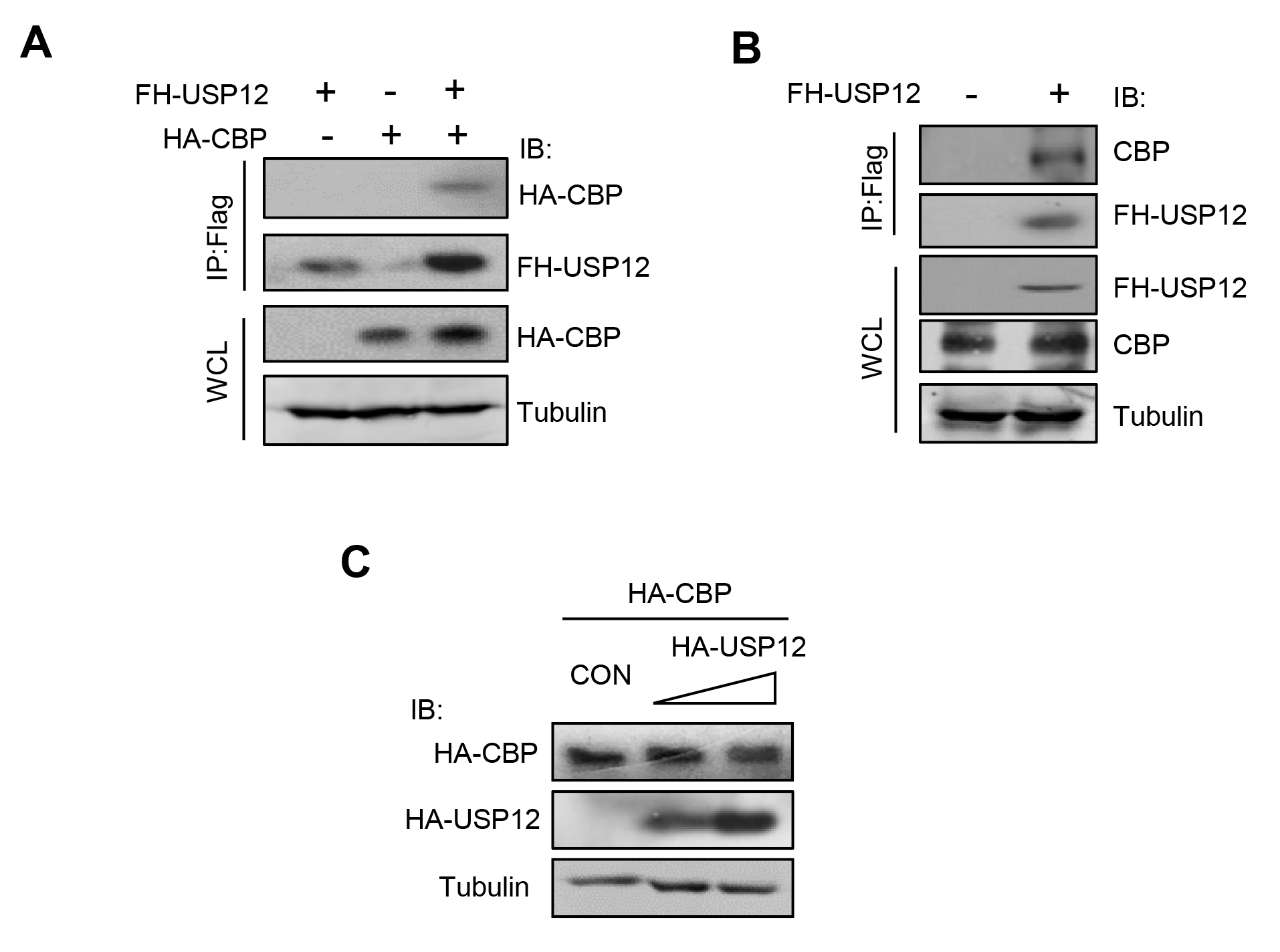

Supplement: S1 Fig — (A) Immunoprecipitation analysis of the interaction between Flag-HA-USP12 (FH-USP12) and HA-CBP in HEK293T cells. (B) Immunoprecipitation analysis of the interaction between CBP and USP12 in HEK293T cells transfected with empty vectors (-) or FH-USP12. (C) Western blot analysis of HA-CBP levels in HEK293T cells cotransfected with either empty vectors (CON) or increasing amounts of HA-USP12, together with HA-CBP. (TIF) [file ppat.1008215.s001.tif]

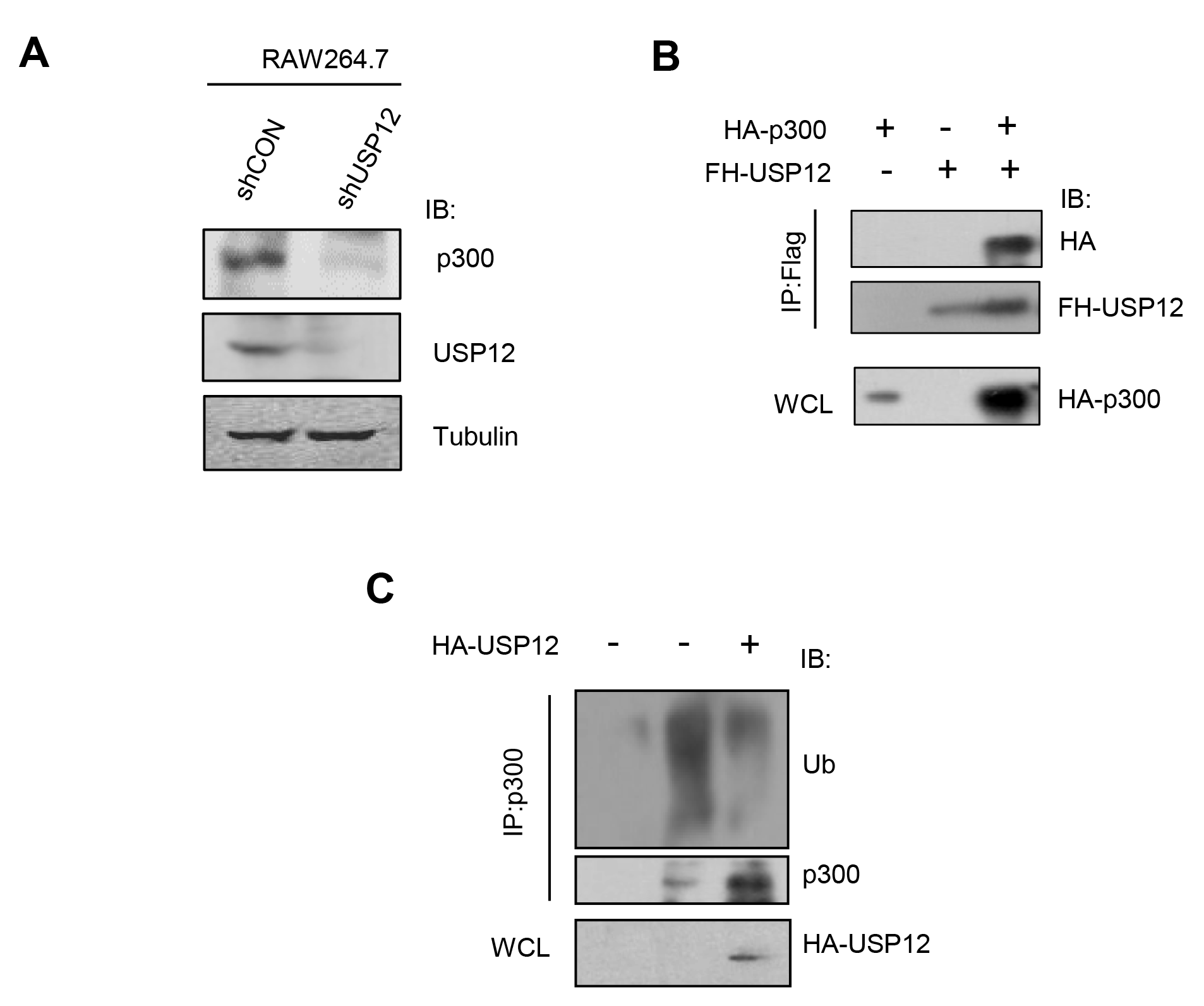

Supplement: S2 Fig — (A) Western blot analysis of p300 protein levels in RAW264.7 cells transfected with either control shRNAs (shCON) or shRNAs against USP12 (shUSP12). (B) Immunoprecipitation analysis of the interaction between FH-USP12 and HA-p300 in HEK293T cells. (C) HEK293T cells transfected with empty vectors (-) or HA-USP12. Cells then were treated with MG132 (10 μM) for 4 h. Immunoprecipitation (IP) and immunoblotting (IB) were performed as indicated. (TIF) [file ppat.1008215.s002.tif]

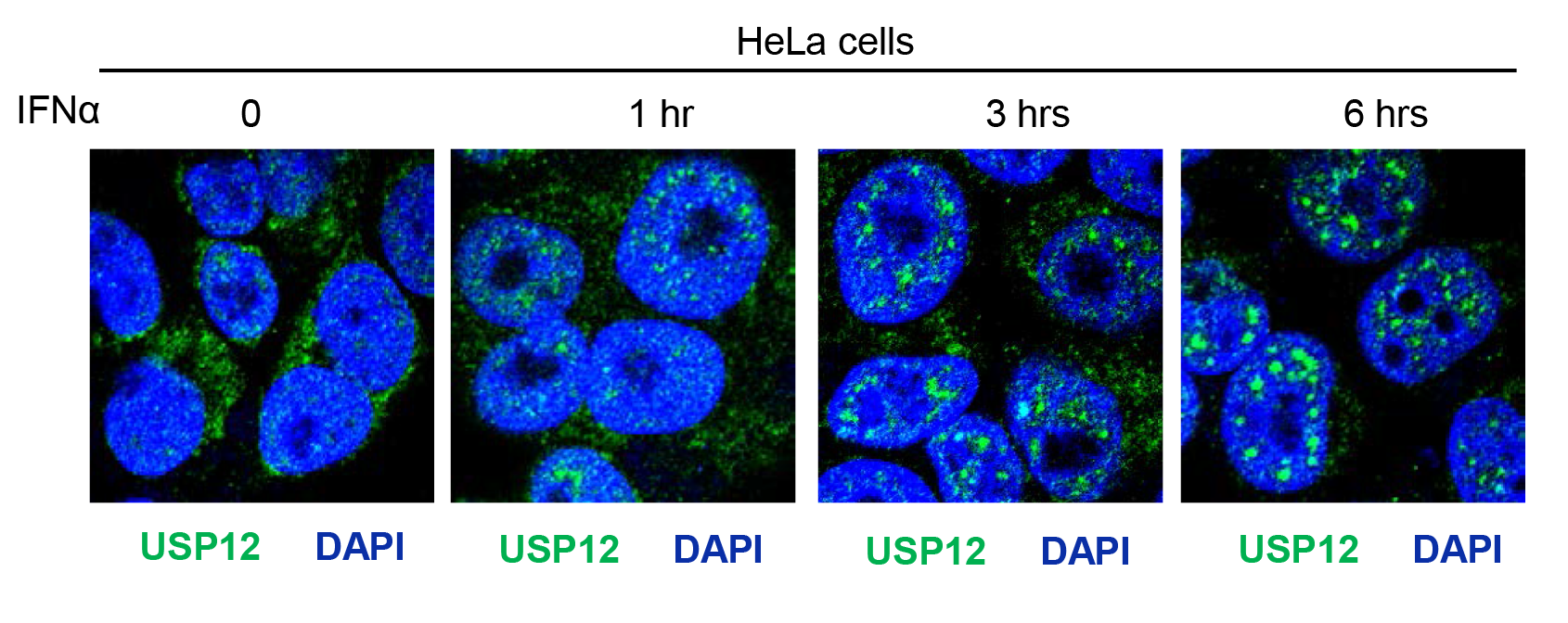

Supplement: S3 Fig — HeLa cells were treated with IFNα (3,000 IU/ml) for 0, 1, 3 and 6 hrs. Cellular USP12 proteins were stained by specific USP12 antibodies, and cell nuclei were stained by DAPI. The fluorescent images were captured with the Nikon A1 confocal microscope. (TIF) [file ppat.1008215.s003.tif]

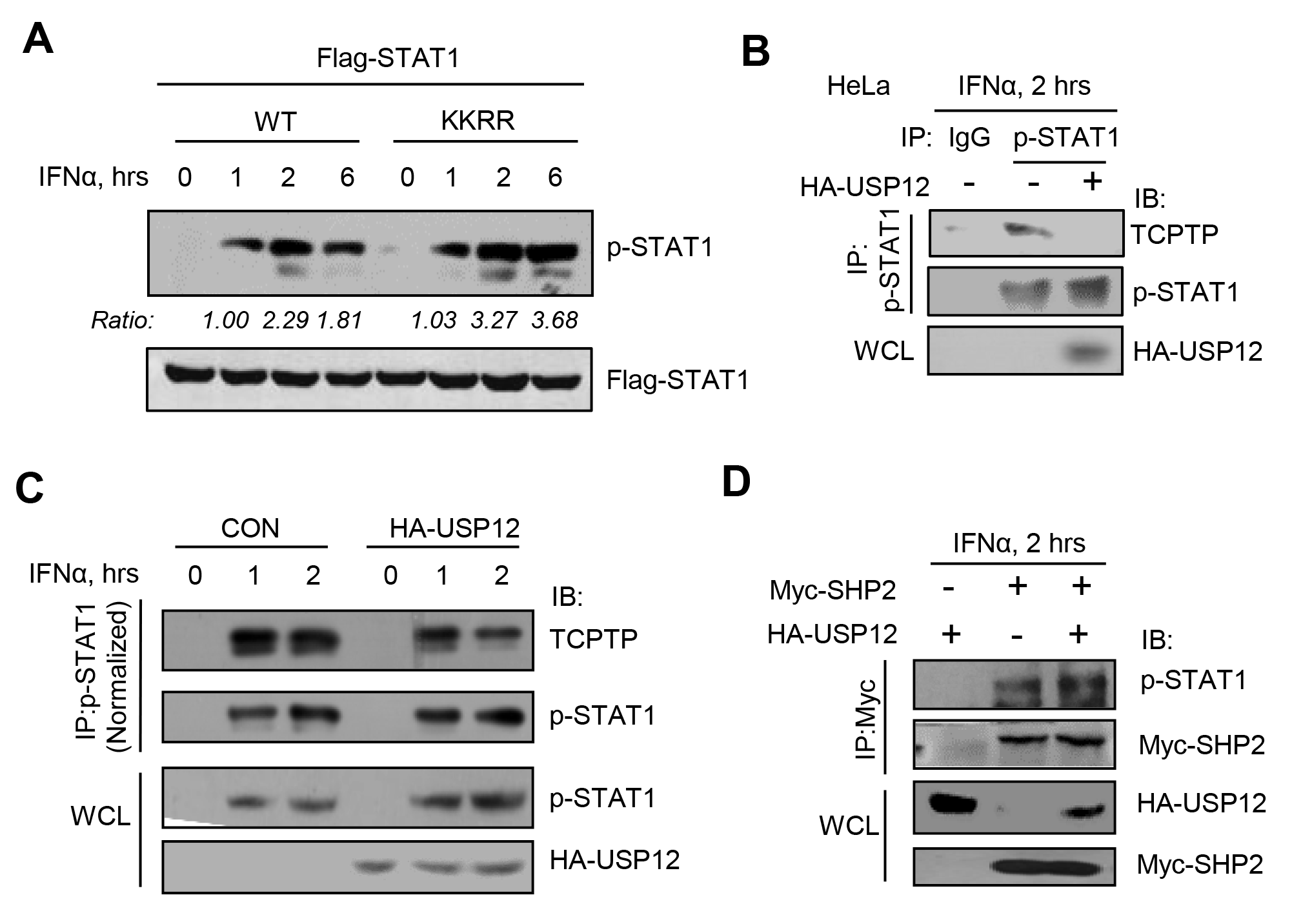

Supplement: S4 Fig — (A) STAT1-deficiency fibroblast cells U3A were transfected with Flag-STAT1-WT or Flag-STAT1-K410R, K413R (KKRR), followed by treatment with IFNα (1,000 IU/ml) as indicated. Phosphorylated STAT1 at Tyr701 (p-STAT1) was analyzed using a specific antibody. (B) Immunoprecipitation analysis of the interaction between p-STAT1 and TCPTP in HeLa cells transfected with HA-USP12 and then treated with IFNα (1,000 IU/ml) for 2 hrs. (C) Immunoprecipitation analysis of the interaction between p-STAT1 and TCPTP in HEK293T cells transfected with HA-USP12 and then treated with IFNα (1,000 IU/ml) for 0, 1, and 2 hrs. (D) Immunoprecipitation analysis of the interaction between p-STAT1 and Myc-SHP2 in HEK293T cells cotransfected with HA-USP12 and (or) Myc-SHP2 and then treated with IFNα (1,000 IU/ml) for 2 hrs. (TIF) [file ppat.1008215.s004.tif]

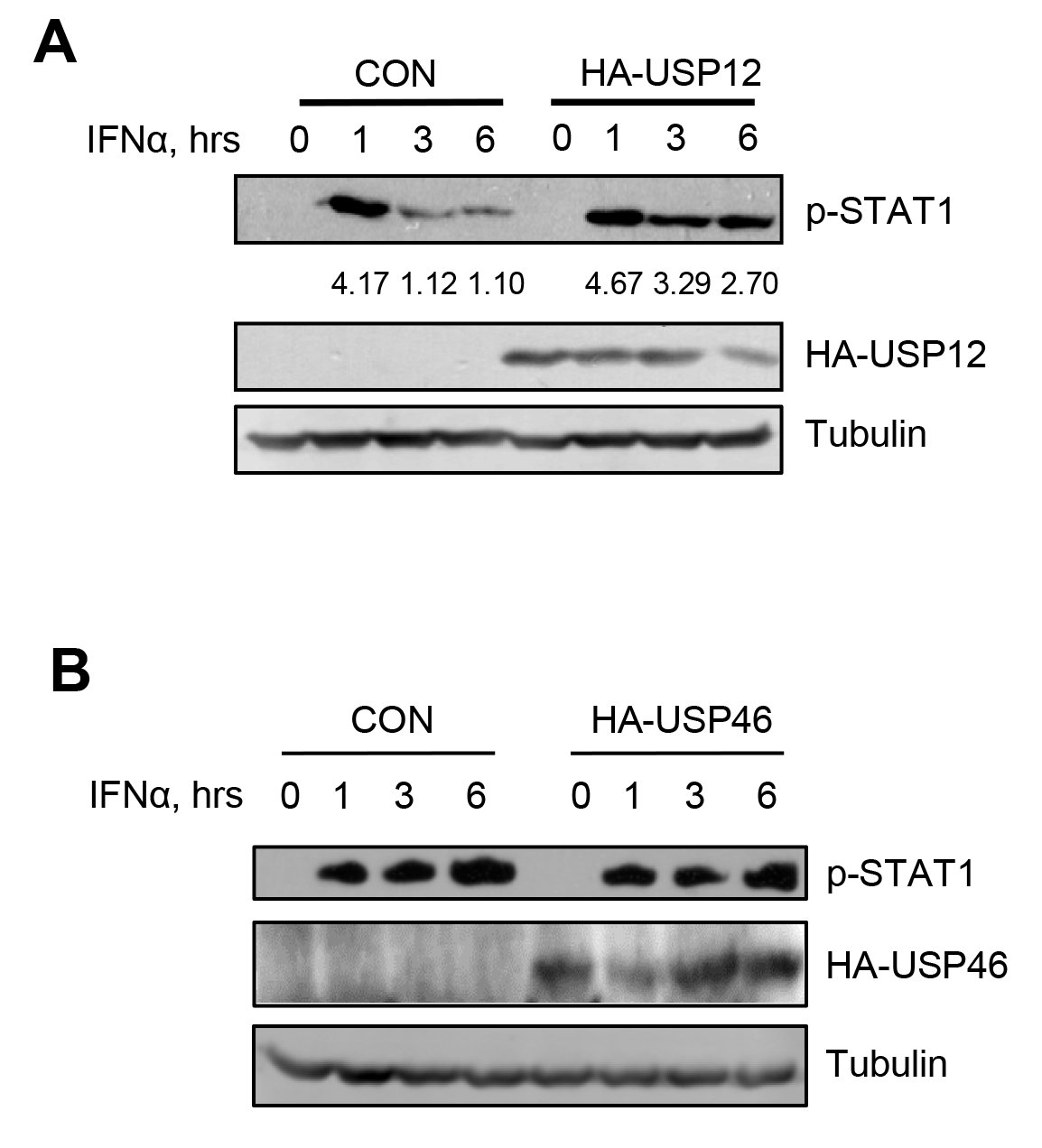

Supplement: S5 Fig — (A) Western blot analysis of p-STAT1 levels in HEK293T cells transfected with HA-USP12 and then treated with IFNα (1,000 IU/ml) for 0, 1, 3, and 6 hrs. (B) Western blot analysis of p-STAT1 levels in HEK293T cells transfected with HA-USP46 and then treated with IFNα (1,000 IU/ml) for 0, 1, 3, and 6 hrs. (TIF) [file ppat.1008215.s005.tif]

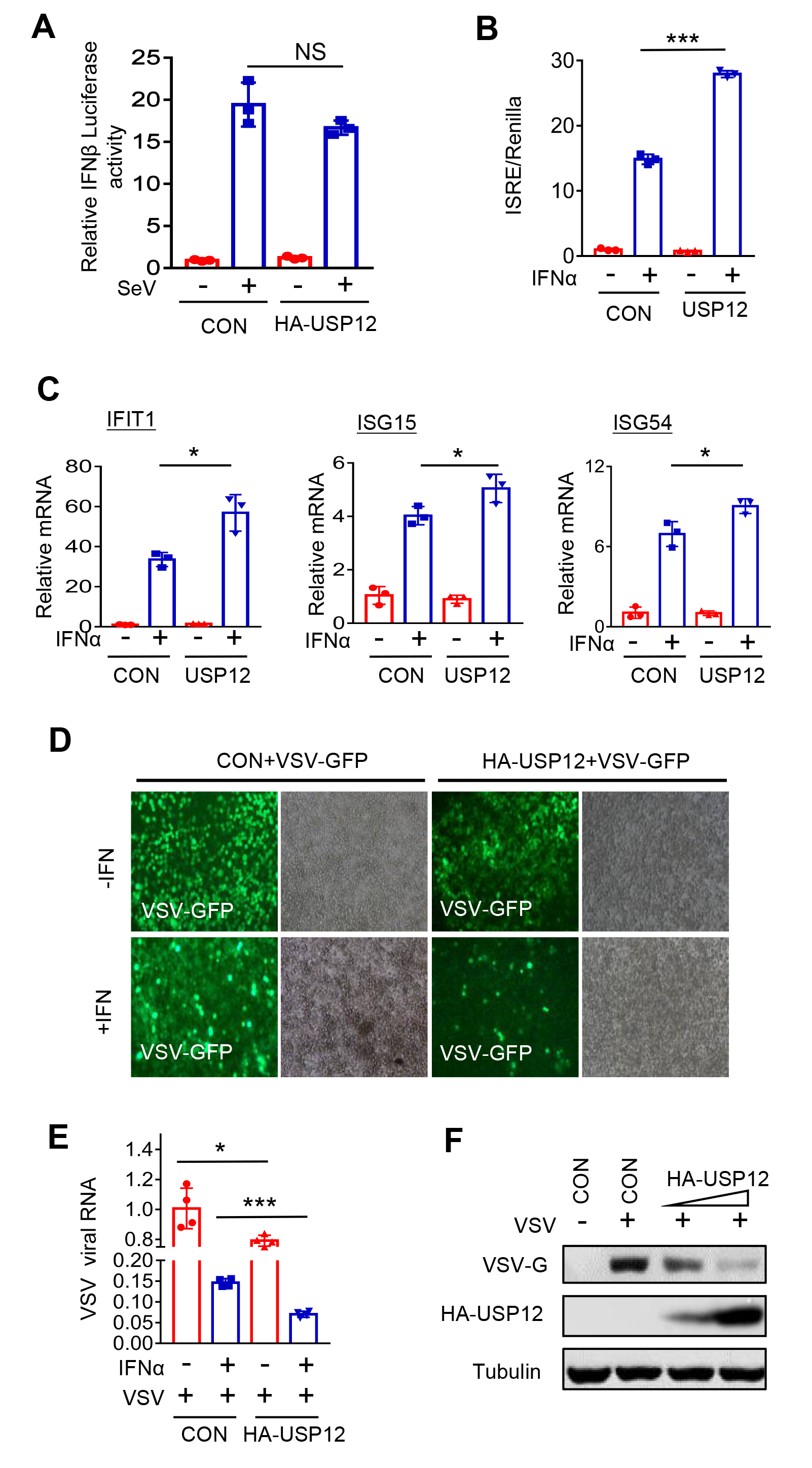

Supplement: S6 Fig — (A) HEK293T cells were transfected with empty vectors (CON) or HA-USP12, together with IFNβ-Luciferase and Renilla. The luciferase activity was measured 20 hrs after SeV (MOI = 0.5) infection. (B) HEK293T cells were transfected with empty vertors (CON) or HA-USP12, together with ISRE-Luc and Renilla. The luciferase activity was measured 20 hrs after IFNα (1,000 IU/ml) treatment. (C) Q-PCR analysis of representative ISGs (IFIT1, ISG15 and ISG54) mRNA levels in HEK293T cells transfected with empty vectors (CON) or HA-USP12, and then treated with IFNα (1,000 IU/ml) for 8 hrs. (D) HEK293T cells transfected with empty vectors (CON) or HA-USP12 were treated with IFNα (50 IU/ml) overnight. Cells were challenged by VSV-GFP (MOI = 0.5). After 24 hrs, VSV-GFP levels were detected by fluorescence. (E) 2fTGH cells transfected with or without HA-USP12 were treated with IFNα (30 IU/ml) overnight, and then cells were challenged by VSV (MOI = 1.0). After 20 hrs, the level of VSV viral RNA was analyzed by Q-PCR. (F) Western blot analysis of VSV-G protein levels in HCT116 cells transfected with HA-USP12 and then challenged with VSV (MOI = 0.5) for 20 hrs. NS, not significant (p>0.05), *p<0.05 and ***p<0.001 (two-tailed unpaired Student’s t-test). Data are shown as mean and s.d. of three biological replicates. (TIF) [file ppat.1008215.s006.tif]
